# Tropic origins, a dispersal model for saprotrophic mushrooms in *Agaricus* section *Minores* with descriptions of sixteen new species

**DOI:** 10.1038/s41598-017-05203-5

**Published:** 2017-07-11

**Authors:** Mao-Qiang He, Jie Chen, Jun-Liang Zhou, Cheewangkoon Ratchadawan, Kevin D. Hyde, Rui-Lin Zhao

**Affiliations:** 1 State key laboratory of Mycology, Institute of Microbiology, Chinese Academy of Sciences, Beijing, 100101 China; 2 Department of Entomology and Plant Pathology, Faculty of Agriculture, Chiang Mai University, Chiang Mai, 50200 Thailand; 3 Institute of Excellence in Fungal Research, Mae Fah Luang University, Chiang Rai, 57100 Thailand; 4 Institute of Microbiology and Beijing Key Laboratory for Forest Pest Control, Beijing Forestry University, Beijing, 100083 China; 5 College of Life Sciences, University of Chinese Academy of Sciences, Huairou District, Beijing, 100408 China

## Abstract

*Agaricus* section *Minores* contains the richest species diversity within the genus. Its Phylogeny is firstly presented by a Maximum Likelihood tree generated through DNA sequences from four gene regions of 91 species. Furthermore, a molecular dating analysis is conducted used those sequences, and it provided the divergence times of the clades within section *Minores*. Study showed section *Minores* has a tropical origin. Four main dispersal routes are proposed: (1) species from South Asia migrated through the Tibetan Plateau and reached Europe ca. 9–13 Ma; (2) species from out of South Asia dispersed to Europe in the earlier time of ca. 22 Ma; (3) species from South Asia dispersed through North Asia to Alaska, and reached West America around ca. 9 Ma; and (4) species from South Asia dispersed south and reached Oceania by at least three invading events about ca. 9, 12 and 16–18 Ma respectively. Those routes excepting the second route coincide with those of ectomycorrhizal mushrooms. To know whether the second route existed in the saprotrophic mushrooms requires further studies, and the fourth route may explain why the secotioid species occurring in Australia are morphologically similar but cluster in different phylogenetic clades. This study also demonstrates a great biodiversity of *A*. section *Minores* in China. Sixteen new species and three new records are introduced from China with morphological descriptions, illustrations, color photographs and phylogenetic analyses.

## Introduction

*Agaricus* L. (Agaricaceae, Agaricales), the type genus of Agaricaceae, contains abundant species distributed across all continents^1, 2^. Many species in this genus are well-known because of their high commercial value, such as *A*. *bisporus* (J.E. Lange) Imbach and *A*. *subrufescens* Peck; both having been commercially cultivated for many years. It was estimated that there are about 200 species of *Agaricus* worldwide^3^. However, the number of species in this genus has increased rapidly since 2008 because new species have been introduced. In 2011, the estimated number of *Agaricus* was 386^1^. To date, *Agaricus* comprises more than 500 species, as numerous new species have been introduced^4–10^.

There have been a series of phylogenetic studies on *Agaricus* since 1999^11^, and these studies contributed to build a more robust phylogenetic framework and related taxonomic system for this genus. A taxonomic system for *Agaricus* comprising three subgenera and eight sections was used for a long time^4, 12, 13^. Some of those sections have been confirmed as monophyletic groups, such as *A*. section *Bivelares* (Kauffman) L.A. Parra^14^, while some others have been shown to be polyphyletic, such as section *Spissicaules* (Heinem.) Kerrigan^13^. A phylogenetic analysis with emphasis on *Agaricus* specimens from tropical areas revealed eleven new clades, mainly from tropical areas besides those eight previously diagnosed sections^1^. As a result of these phylogenetic discoveries, several sections have been proposed, such as sections *Nigrobrunnescentes* K.R. Peterson, Desjardin & Hemmes. and *Brunneopicti* Heinem^15, 16^, or established, such as sections *Rarolentes* Kerrigan and *Subrutilescentes* Kerrigan^7^. The most recent study combined multi-gene phylogeny, morphology and divergence times, established a comprehensive taxonomic system for *Agaricus* ^9^. In that study, *Agaricus* was segregated into five subgenera and 20 sections, subgenus *Minores* was established and comprised three sections.

The epithet “*Minores”* was established in 1874 to accommodate a small group of fungi with small basidiomes^17^. Morphological and biochemical examination have shown that species in this section always have a strong positive KOH reaction and Schäffer’s reaction, a single annulus, basidiomes which are flavescent on cutting and bruising, an odour of almond or anise. Most of the species in this section are saprobic and edible^4, 9^. Historically, species of section *Minores* were known by their small basidiomes, but recent studies have shown that species from this section can also have large-sized basidiomes^8, 18, 19^, which provides the possibility of developing some species as cultivated mushrooms for food^10, 18^.

The number of species in *A*. section *Minores* has been underestimated. Less than 20 species were known before the early 21^st^ century^12, 20, 21^. However, recently, 21 species from Europe^4^ and 38 species from Greater Mekong Subregion^10^ have been recognized. It was hypothesized that there are at least 200 species in this section worldwide^10^.

The origin and dispersal of fungi has been of great interest to mycologists. Fungi can be dispersed by human activities^22, 23^, insects (insect associated fungi)^24^, and also other factors like climate and geographical history^25–27^. Phylogeographical evidence suggests ectomycorrhizal mushrooms dispersed via overland routes, because of their obligate symbiotic associations with woody plants^28–30^, especially *Boletus* ^31, 32^, *Chroogomphus* ^33^, *Amanita* ^25, 34^, *Sparassis* and *Megacollybia* ^35–40^. However, investigations on saprotrophic fungi origins and dispersal have rarely been studied^41^, especially among mushrooms.

In this study, based on our *Agaricus* project over five years in China and previous published data of the section *Minores* from other continents and countries^1, 4, 10, 18, 42–45^, we address the origin and dispersal of the saprotrophic mushroom genus *Agaricus* section *Minores* based on timing of evolutionary events. The species new to science are described in the taxonomic part of this paper with phylogenic analyses and morphological characteristics.

## Results and Discussion

### Phylogenetic analysis

A totally of 154 assembled multi-gene sequences were included for phylogenetic reconstruction, representing 97 species from the subgenus *Minores*, including 91 species of the section *Minores*, three species of the section *Leucocarpi*, three species of unnamed section 1; one species of subgenus *Minoriopsis* and the outgroup taxon *A*. *campestris* L. There are 2675 bp (base pairs) in the final alignment of each assembled sequence, of which 744 characters are from LSU, 541 characters from tef1-α, 776 characters from rpb2 and 614 characters from ITS.

**Figure 1:**
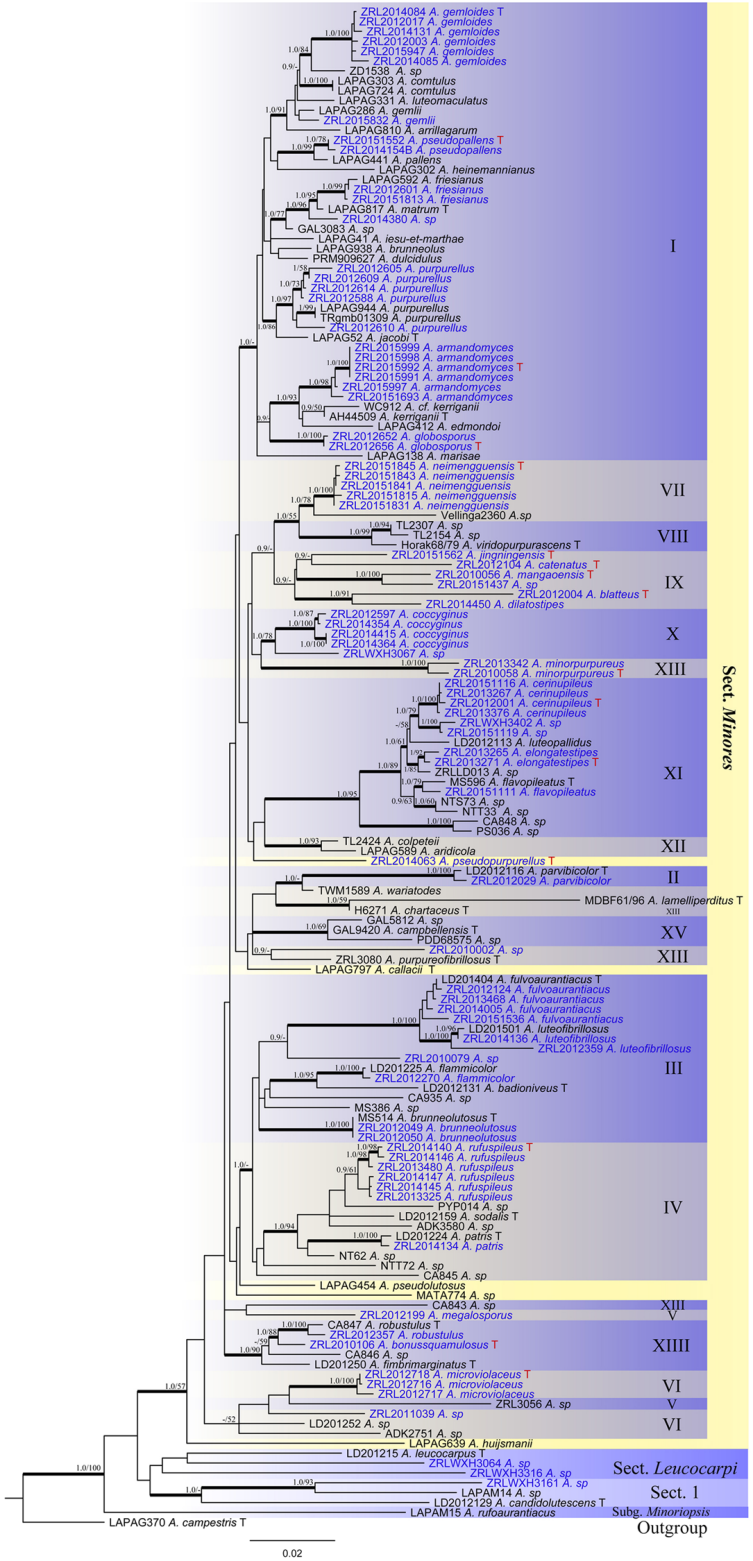
Maximum Likelihood (ML) tree of *Agaricus* section *Minores* based on LSU, tef1-α, rpb2 and ITS sequences with the outgroup *Agaricus campestris*. The Bayesian posterior probabilities and bootstrap support values more than 0.9/50% (PP/BS) are indicated at the nodes. The branches in Bold mean the related PP > 0.95. Sequences produced from this study are in blue. “T” refers to sequences from type specimen, and “T” in red refers to the sequences from type specimen and new to science from this study.

**Figure 2:**
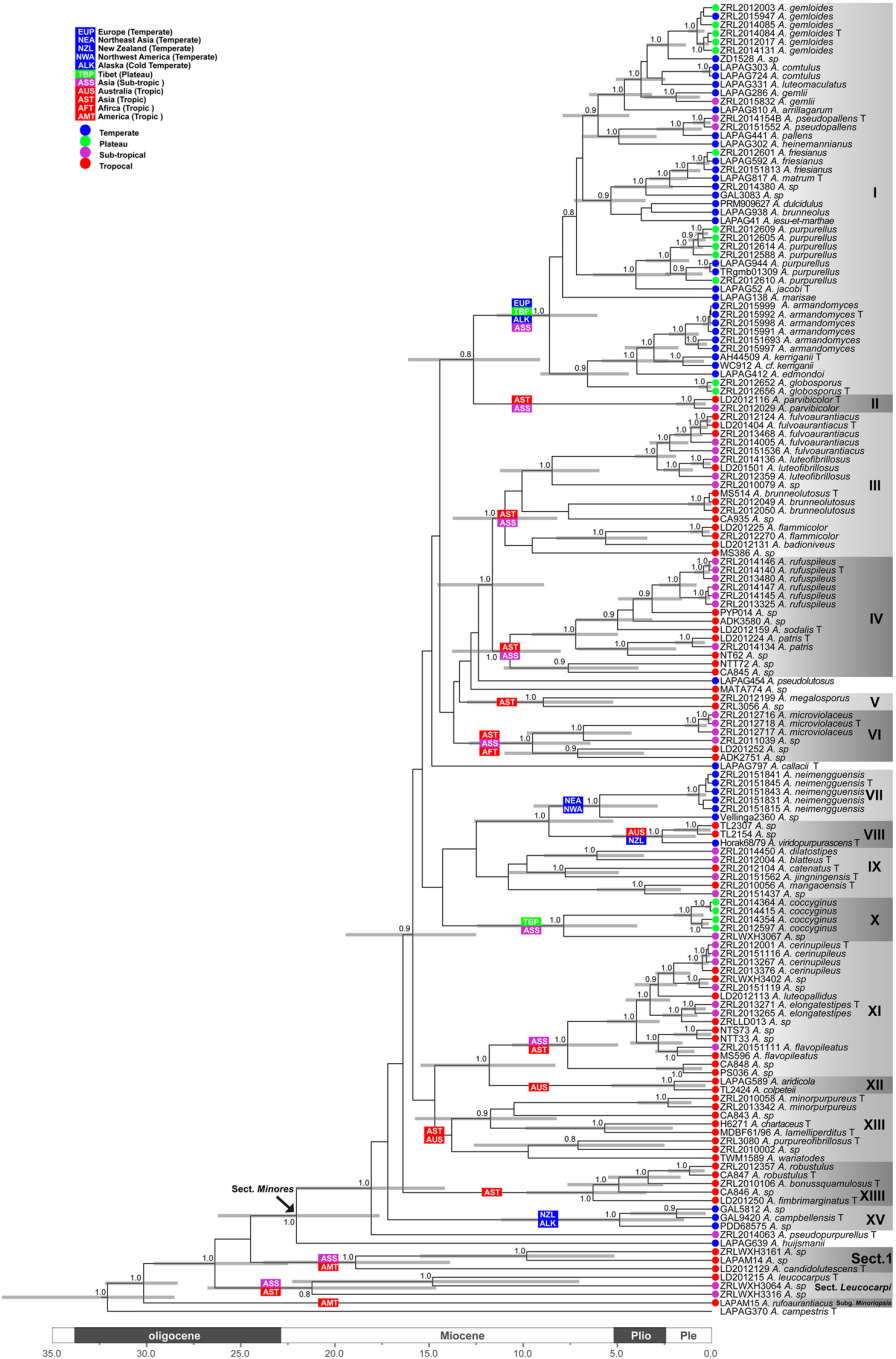
Maximium Clade Credibility (MCC) tree of *Agaricus* subgenus *Minores* based on LSU, tef1-α, rpb2 and ITS sequences with the outgroup *Agaricus campestris*. Posterior probability equal and above 0.8 are indicated at the nodes. The 95% highest posterior density (HPD) of divergence time estimation is marked by horizontal bars.

Multi-gene trees generated from ML, MP and Bayesian analyses yielded highly similar topologies with some ungrouped taxa. The ML tree is shown in Fig. 1. In this tree, section *Minores* are supported by 57% BS and 1.0 PP values, separated from other two sections *Leucocarpi* and unnamed section 1 (Fig. 1) under the clade represented as subgenus *Minores*. Within section *Minores*, 15 clades are recognized in this study and named as Clades I–XV mainly based on the molecular dating analysis (Fig. 2), and those 15 clades also reflects in the ML tree (Fig. 1). In the ML tree (Fig. 1), the clades II, VII, VIII, X, XI, XII, XIIII, and XV are well support with the PP/BS of 0.9–1/69–100%; clade I has a support of 1.0/- PP/BS; however the clades III–VI, IX and XIII are not supported because of failed to form the monophyletic groups or low statistical support. The proposed new species in this study are well-supported with values of 0.9/- to 1.0/100 PP/BS.

### Divergence time and phylogeography analysis

The MCC tree is shown in Fig. 2. The topologies of MCC are generally similar with Fig. 1 with those differences: (1) The unsupported or not well-supported clades in ML analysis (Clades I, III–VI) are fully supported in the molecular dating analysis (Fig. 2) which generated by increasing the generations of Monte Carlo Markov Chains. (2) Clades IX and XIII are still lacking the statistical support in both analyses (Figs 1–2). Clades XIII comprises seven species (*A*. *minorpurpureus*, *A*. sp/CA843, *A*. *chartaceus*, *A*. *lamelliperditus*, *A*. *purpureofibrillosus*, *A*. sp/*ZRL2010002* and *A*. *wariatodes*) in the MCC tree with the PP values less than 0.8 (Fig. 2), but failed to group together in the ML tree (Fig. 1). Clade IV comprises eight species as a monophyletic group in both analyses, however with a statistical values of less than 0.8/50% PP/BS. All proposed new species and known species are well-supported in the phylogenetic and BEAST analyses.

Based on the related isolated geographic origin, those 15 clades in section *Minores* are named as Clades I–XV. Generally, these clades (named as Clades I–VIII, X–XII, and XIIII–XV) have good support of 0.8–1.0 PP value; and Clades IX and XIII have poor statistical support of 0.7 and 0.4 PP values respectively. There are several ungrouped species: *Agaricus huijsmanii* Courtec., *A*. *pseudopurpurellus* M.Q. He & R.L. Zhao, *A*. *callacii* L.A. Parra, R. Iglesias, Fdez.-Vic. & Oyarzabal, *A*. *pseudolutosus* (G. Moreno, Esteve-Rav., Illana & Heykoop) G. Moreno, L.A. Parra, Esteve-Rav. & Heykoop, and *A*. sp./MATA774.

Combined with the different climate types (temperate, sub-tropical and tropical) and relative isolated positions (Tibetan Plateau), we named the 15 phylogenetic clades as 11 types, which are abbreviated as EUP (Europe, temperate); NEA (Northeast Asia, temperate), NZL (New Zealand, temperate), NWA (Northwest America, temperate), ALK (Alaska, cold temperate), TBP (Tibet, plateau), AMT (America tropic), ASS (Asia, sub-tropic), AUS (Australia, tropic), AST (Asia, tropic) and AFT (Africa, tropic). We use mean stem age to represent the divergence times^9^ of those clades. The established divergence times of those clades and ungrouped species are presented in Table 1 and Fig. 2.

**Table 1:** Mean of stem ages of clades and ungrouped species from section *Minores*.

Clades and ungrouped species	Mean of stem age (Ma)
I	13
II	13
III	12
IV	12
V	ca. 9–16
VI	ca. 9–16
VII	9
VIII	9
IX	–
X	ca. 8–16
XI	12
XII	12
XIII	–
XIIII	ca.16–18
XV	ca.16–18
*A*. *huijsmanii*	22
*A*. *pseudopurpurellus*	18
*A callacii*	ca.16
*A*. *pseudolutosus*	ca.16
*A*. sp./MATA774	ca.16

## Conclusions

### Phylogeny of *Agaricus* section *Minores*

*Agaricus* section *Minores* belongs to *A*. subgenus *Minores* in the standardized taxonomic system which was established by evidence from analysis of combined multi-gene sequence data and divergence time analyses^9^. Twenty-one recognized species of this section have been well-documented in Europe^4^. Thirty-eight species from this section were reported from the Greater Mekong Subregion^10^ (GMS: a region around the Mekong River basin in Southeast Asia, which includes Cambodia, Laos, Myanmar, Thailand, Vietnam, and Yunnan Province of China). In this study, we include all known species with molecular data, plus 105 Chinese samples and present a phylogeny of subgenus *Minores*, including 91 phylogenetic species of section *Minores*. The results using morphology and multi-gene sequences analysis resolved 58 species in this section, 38 of which can be found in China, including 16 species are new to science and three species are new records for China (for details see Taxonomy part).

Our phylogenetic result shows a highly similar topology with those of Chen *et al*.’s work^10^. Chen *et al*. recognized eleven clades, which are identical to our clades I, V, VI, X, XI, XIIII and XV. The exceptions are clade IX which is a new clade in this study, and clades II, III, IV, VII, VIII, XII and XIII failed to form monophyletic lineages or had the statistical support of less than 0.8 PP. Morphological traits of those clades in section *Minores* vary and tend to be overlap^10^. However, with larger sampling and statistics of the morphology features in every species (see Table 2), we found that the fibrils color of the pilei is an informative phylogenetic trait in section *Minores*. For example, reddish-brown (such as pinkish-brown, and purplish-brown) is common in most species of this section. Species with yellowish-brown fibrils on the pileus surface are only found in clades III and IX, which are specifically from tropical and subtropical.

**Table 2:** Main morphological features of species in *Agaricus* sect. *Minores*, *means new species described in this study.

Species	Clade	Cap size	Fibrils (Scales) Color	Basidiospore	Cheilocystidia
*A*. *gemloides* ^5^	I	14–36 mm	reddish-brown	4.7 ± 0.2 × 3.6 ± 0.1 μm, Q_m_ = 1.3 ± 0.1	hyaline, ellipsoid, globose, capitate with long narrow stipe
*A*. *comtulus* ^4^	I	15–60 mm	reddish-brown, light ochre	4.8 × 3.4 μm, Q_m_ = 1.4 (LAPAG303)	variable, simple, septate at base, absent
*A*. *gemlii* ^4^	I	25–60 mm	reddish-purple	5.6 × 3.8 μm, Q_m_ = 1.5	hyaline, simple, septate at base, clavate, capitate with long narrow stipe
*A*. *luteomaculatus* ^4^	I	30–57 mm	ochraceous-brown, purplish	6.0 × 4.1 μm, Q_m_ = 1.5	hyaline, with yellow pigment, simple, catenulate
*A*. *arrillagarum* ^4^	I	25–60 mm	reddish-purple	5.0 × 3.7 μm, Q_m_ = 1.4	rare, hyaline, simple, broadly clavate, pyriform, capitate with long narrow stipe
*A*. pseudopallen*	I	23–38 mm	purplish-red	5.4 ± 0.2 × 3.3 ± 0.1, μm Q_m_ = 1.6 ± 0.1	absent
*A*. *pallens* ^4^	I	23–54 mm	reddish-purple	4.0 × 3.2, μm Q_m_ = 1.4	hyaline, simple, septum at base, multiseptum
*A*. *friesianus* ^4^	I	30–67 mm	reddish-purple	4.9 × 3.3, μm Q_m_ = 1.4	hyaline, brown, simple, septum at base
*A*. *heinemannianus* ^4^	I	24–60 mm	reddish-brown	6.1 × 4.4, μm Q_m_ = 1.4	abundance, hyaline, light brown, septate, catenulate, simple
*A*. *matrum* ^4^	I	15–46 mm	reddish-purple, pink	4.9 × 3.4, μm Q_m_ = 1.5	abundant, hyaline, simple, clavate, pyriform, globose, capitate with long narrow stipe
*A*. *brunneolus* ^4^	I	30–110 mm	reddish, reddish-purple	5.4 × 3.8, μm Q_m_ = 1.4	abundant, simple, septum at base
*A*. *iesu-et-marthae* ^4^	I	30–80 mm	reddish-purple, reddish-brown	6.1 × 4.3, μm Q_m_ = 1.4 (LAPAG33)	hyaline, with brown pigment, simple
*A*. *dulcidulus* ^4^	I	80 mm	pinkish, reddish-pink	3.4 × 3.0, μm Q_m_ = 1.4	hyaline, simple, septum at base
*A*. *purpurellus* ^4^	I	20–50 mm	purplish-red	5.2 × 4.0, μm Q_m_ = 1.3	abundant, simple, septum at base, clavate, capitate with long narrow stipe
*A*. *jacobi* ^4^	I	30–75 mm	reddish-pink, reddish-purple	5.2 × 4.0, μm Q_m_ = 1.4	abundant, hyaline, light brown, multiseptate
*A*. armandomyces*	I	16–42 mm	brown, yellowish-brown	4.9 ± 0.2 × 3.4 ± 0.1, μm Q_m_ = 1.4 ± 0.0	simple, pyriform, septum at base
*A*. *kerriganii* ^4^	I	30–60 mm	reddish-purple, reddish-pink	5.0 × 3.4, μm Q_m_ = 1.4	abundant, mutiseptum
*A*. *edmondoi* ^4^	I	20–60 mm	reddish-brown	4.8 × 3.3, μm Q_m_ = 1.5	abundant, hyaline, simple, clavate, pyriform, globose, capitate with long narrow stipe
*A*. globosporus*	I	11–62 mm	reddish-brown	4.2–5.7 × 3.9–4.6, μm Q_m_ = 1.2 ± 0.1	simple, clavate
*A*. *marisae* ^4^	I	25–44 mm	reddish-brown	6.2 × 4.1 μm, Q_m_ = 1.5	abundant, hyaline, light brown, septate, catenulate
*A*. *parvibicolor* ^19^	II	15–40 mm	reddish-brown, violet brown	5.2 × 3.3 μm, Q_m_ = 1.6	hyaline, abundant, simple, broadly clavate, pyriform, capitate with long narrow stipe
*A*. *luteofibrillosus* ^8^	III	35–94 mm	yellowish-brown	5.8 ± 0.4 × 3.4 ± 0.2 μm, Qm = 1.7 ± 0.1	globose, clavate, pyriform, capitate with long narrow stipe
*A*. *fulvoaurantiacus* ^10^	III	37–70 mm	brownish-yellow, brownish-orange	5.8 × 3.8 μm, Q_m_ = 1.51 ± 0.01	abundant, simple, pyriform, broadly clavate, capitate with long narrow stipe
*A*. *flammicolor* ^10^	III	40–70 mm	bright orange	4.9 × 2.9 μm, Q_m_ = 1.69 ± 0.04	abundant, simple, pyriform, broadly clavate, capitate with long narrow stipe, with yellow pigment
*A*. *badioniveus* ^10^	III	35 mm	yellowish-brown	5.6 ± 0.12 × 3.3 ± 0.11 μm, Q_m_ = 1.67 ± 0.01	abundant, simple, pyriform, narrowly clavate, with yellowish pigment
*A*. *brunneolutosus* ^10^	III	55–85 mm	brown	4.3 × 2.9 μm, Q_m_ = 1.48 ± 0.03	abundant, simple, pyriform, broadly clavate
*A*. rufuspileus*	IV	49–60 mm	brown, reddish-brown	5.8 ± 0.3 × 3.7 ± 0.2 μm, Q_m_ = 1.6 ± 0.1	broadly ellipsoid, globose, broadly clavate, with yellow pigment
*A*. *sodalis* ^19^	IV	42–90 mm	violet brown	5.4 × 3.6 μm, Q_m_ = 1.5	abundant, simple, broadly clavate, pyriform, capitate with long narrow stipe, with yellow pigment
*A*. *patris* ^10^	IV	45–50 mm	reddish brown, purplish brown	6 ± 0.16 × 3.7 ± 0.15 μm, Q_m_ = 1.58 ± 0.01	simple, clavate, broadly clavate, capitate with long narrow stipe
*A*. *megalosporus* ^18^	V	35–110 mm	purplish-brown, brown	6.0 ± 1 × 3.5 ± 0.6 μm, Q_m_ = 1.6 ± 0.6	hyaline, broadly clavate, pyriform with cylindrical base
*A*. microviolaceus*	VI	18–26 mm	purple, reddish-brown	5.2 ± 0.4 × 3.3 ± 0.2 μm, Q_m_ = 1.6 ± 0.1	hyaline, clavate, broadly clavate, with yellow pigment
*A*. neimengguensis*	VII	25–71 mm	yellowish-brown, reddish-brown	5.5 ± 0.3 × 3.8 ± 0.2 μm, Q_m_ = 1.5 ± 0.0	hyaline, ellipsoid, clavate,broadly clavate, with yellow pigment
*A*. *viridopurpurascens* ^66^	VIII	50 mm	brown	4.8–6.3 × 3.5–4.1 μm	globose, catenulate, yellow
*A*. dilatostipes*	IX	44–110 mm	reddish brown	5.1 ± 0.3 × 3.3 ± 0.2 μm, Q_m = _1.6 ± 0.1	simple, hyaline, clavate, broadly clavate, capitate with long narrow stipe
*A*. blatteus*	IX	13–28 mm	dark purple	4.5 ± 0.2 × 3.3 ± 0.1 μm, Q_m = _1.4 ± 0.1	pyriform, broad clavate, ellipsoid, with yellow pigment
*A*. mangaoensis*	IX	22–30 mm	brown, dark brown	5.5 ± 0.3 × 3.6 ± 0.2 μm, Q_m_ = 1.5 ± 0.1	hyaline, clavate, broadly clavate, yellow pigment
*A*. jingningensis*	IX	32–78 mm	reddish-brown, purplish-brown	4.8 ± 0.3 × 3.3 ± 0.1 μm, Q_m_ = 1.4 ± 0.1	clavate, ellipsoid, septum at base, with yellow pigment
*A*. catenatus*	IX	50 mm	light brown, purplish-red	5.1 ± 0.2 × 2.7 ± 0.1 μm, Q_m_ = 1.4 ± 0.1	simple, clavate, globose, ellipsoid, catenulate, with yellow pigment
*A*. *coccyginus* ^8^	X	35–110 mm	purplish-red, brown	6.0 ± 0.3 × 3.8 ± 0.2 μm, Q_m = _1.6 ± 0.1	pyriform, clavate, oblong, capitate with long narrow stipe, with yellow pigment
*A*. cerinupileus*	XI	70–90 mm	yellowish brown	6.0 ± 0.3 × 3.5 ± 0.2 μm, Q_m_ = 1.6 ± 0.1	capitate with long narrow stipe, globose, clavate, broadly clavate, hyaline, with yellow pigment
*A*. *luteopallidus* ^10^	XI	30–60 mm	pallid yellow, brownish yellow	5.4 ± 0.4 × 3.6 ± 0.3 μm, Q_m = _1.52 ± 0.02	abundant, simple, globose, pyriform, capitate with long narrow stipe, with yellowish pigment
*A*. elongatestipes*	XI	55–58 mm	yellowish brown	5.0 ± 0.2 × 3.3 ± 0.2 μm, Q_m_ = 1.5 ± 0.1	capitate with long narrow stipe, globose, ellipsoid, broadly clavate
*A*. *flavopileatus* ^10^	XI	40–60 mm	grayish yellow, yellow ochre	4.8 ± 0.13 × 2.9 ± 0.15 μm, Q_m_ = 1.7 ± 0.1	abundant, simple, pyriform, broadly clavate, capitate with long narrow stipe, with yellowish pigments
*A*. *aridicola* ^67^	XII	15–40 mm	—	—	—
*A*. *colpeteii* ^45^	XII	8–33 mm	grayish, silvery white	6.9 ± 0.5 × 5.9 ± 0.35 μm, Q_m_ = 1.0–1.3	—
*A*. *chartaceus* ^44^	XIII	12–27 mm	white, pale cream	7.2 × 5.9 μm, Q = 1.0–1.3	—
*A*. *lamelliperditus* ^45^	XIII	10–30 mm	white, pale cream	7.0–8.5 × 5.0–6.5 μm, Q = 1.0–1.3	—
*A*. minorpurpureus*	XIII	16–18 mm	reddish-brown, purplish-brown	5.0 ± 0.2 × 3.3 ± 0.2 μm, Q_m_ = 1.5 ± 0.1	hyaline, clavate
*A*. *wariatodes* ^44^	XIII	8–18 mm	cream, pinkish huff	7.2 × 6.4 μm, Q = 1.0–1.3	—
*A*. *purpureofibrillosus* ^10^	XIII	20–30 mm	purplish-brown	4.9 ± 0.12 × 2.9 ± 0.14 μm, Q_m_ = 1.69 ± 0.02	abundant, simple, pyriform, capitate with long narrow stipe, broadly clavate, with yellowish pigments
*A*. *robustulus* ^10^	XIIII	20–60 (−8.5) mm	reddish brown, dark golden brown	5.8 ± 0.25 × 3.7 ± 0.16 μm, Q_m_ = 1.56 ± 0.11	simple, ovoid, pyriform, broadly clavate with a thin base, with yellowish pigments
*A*. bonussquamulosus*	XIIII	55 mm	brown	5.8 ± 0.3 × 3.5 ± 0.2 μm, Q_m_ = 1.7 ± 0.1	hyaline, pyriform, broadly clavate
*A*. *fimbrimarginatus* ^10^	XIIII	40 mm	purplish	4.7 ± 0.11 × 3.2 ± 0.09 μm, Q_m = _1.5 ± 0.1	simple, pyriform, broadly clavate, with yellowish pigments
*A*. *campbellensi* ^68^	XV	30–50 mm	clay brown, mustard brown	7–8.8 × 4–4.5 μm, Q = 1.69 ± 0.22	—
*A*. *pseudolutosus* ^4^	unknown	25–66 mm	reddish-purple, reddish-brown	6.4 × 4.8 μm, Q_m_ = 1.3	abundant, variable, hyaline
*A*. pseudopurpurellus*	unknown	20–30 mm	purple	4.8 ± 0.2 × 3.3 ± 0.2 μm, Q_m_ = 1.5 ± 0.1	absent
*A*. *callacii* ^4^	unknown	6–22 mm	ochraceous-brown	6.2 × 4.9 μm, Q = 1.3	rare, clavate
*A*. *huijsmanii* ^4^	unknown	14–40 mm	white, ochraceous	5.0 × 3.4 μm, Q = 1.5	abundant, hyaline, simple, broadly clavate, pyriform, capitate with long narrow stipe, spherical

### Phylogeography of *Agaricus* section *Minores*

Species of *Agaricus* section *Minores* have a worldwide distribution^1, 4, 10, 44^. The evolutionary history of section *Minores* and phylogenetically closely related sections have been thought to be heavily affected by geographical and climatic factors^1, 10^. In this study, we include a large number of species of this section from different geographic area; these geographic areas are defined as 11 types (in Fig. 2, coded as EUP, NEA, NZL, NWA, ALK, TBP, ASS, AUS, AST, AFT and AMT) based on discrete units marked by the present limits of dispersal. To address the dispersal routes of species in *A*. section *Minores*, we defined the 15 phylogenetic clades (I–XV, in Figs 1–2) with its geographical origin (Fig. 2). Both phylogeny and molecular dating analysis (Figs 1–2) indicate that the distribution of same species or clades have the same or similar climatic distributions. On the contrary the different species or clades are generally in their isolated geography areas respectively. Combined with divergence times and geographic origin of those clades and those of ungrouped species could speculate the origin and dispersal routes of section *Minores*.

This study shows species in the basal clade of subgenus *Minores* are originated from tropic Africa, America and Asia, such as *A*. *rufoaurantiacus* Heinem., *A*. *candidolutescens* L.J. Chen & R.L. Zhao, and *A*. *leucocarpus* L.J. Chen, Callac, R.L. Zhao & K.D. Hyde, which agree with the previous study^1, 9, 10^. For the speciation time, tropical species originate between 1.98 to 8.93 Ma, which is generally older than most species origin from sub-tropical and temperate areas (forming at 1.5 to 7.7 Ma and 1.27 to 5.94 Ma respectively). Hence we conclude species section *Minores* is a tropic origin group.

**Figure 3:**
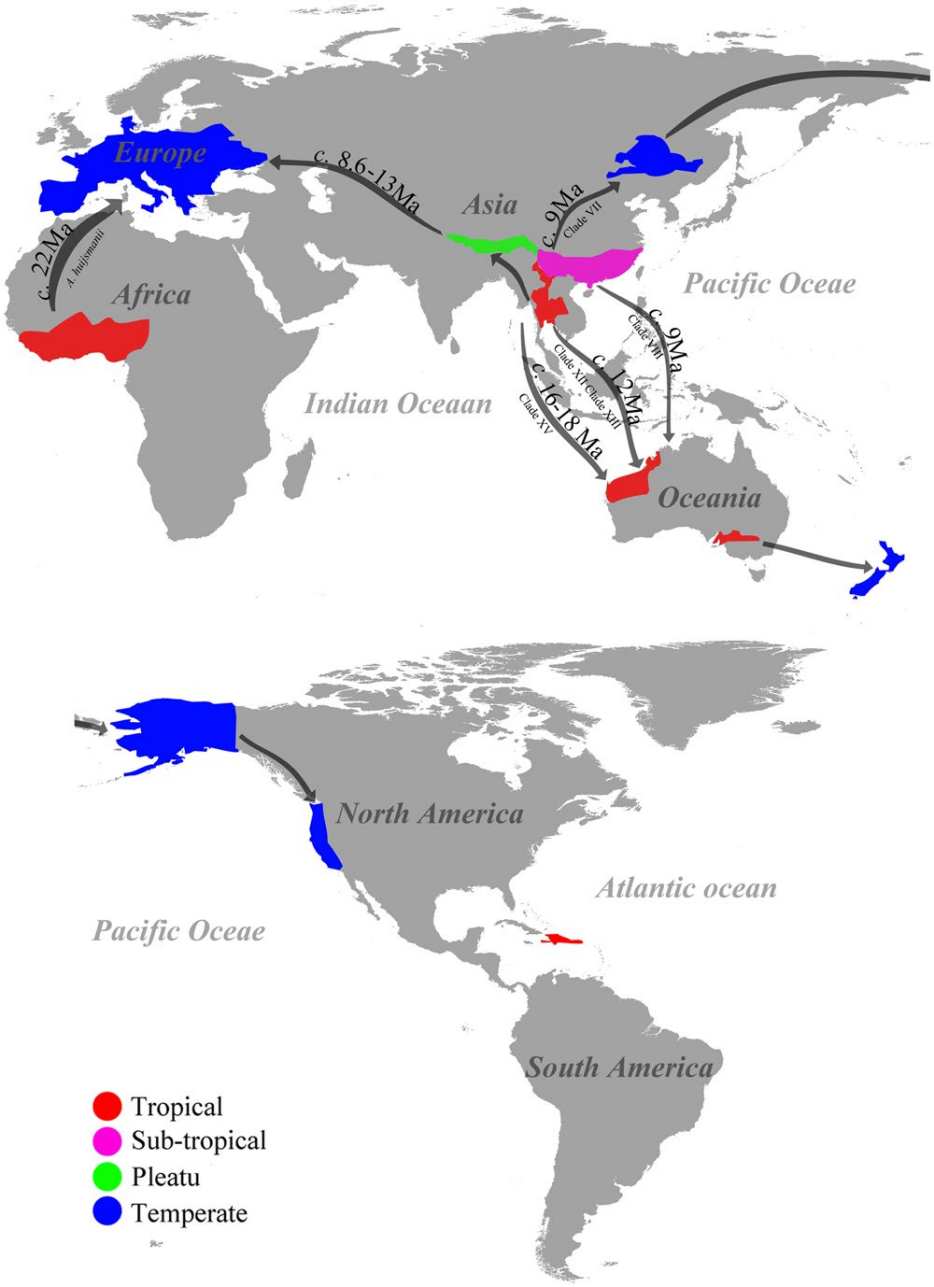
The hypothesized dispersal routines of species of *Agaricus* section *Minores*. The world map delimits the distribution areas of species involved in this study, and arrows indicate the major biogeographical events. Map was generated by ArcGIS v10.1 (http://esri.com/arcgis).

Even several clades (IX and XIII) failed to establish divergence times due to the phylogenetic support values are less than 0.8 PP, the rest 13 clades are successfully dated. We then hypothesized that there are four routes which species section *Minores* spread to North America, Oceania and Europe from their tropic origin areas (Fig. 3). We speculate that species of section *Minores* from Europe are transmigrated through two routes in different times. This conclusion is based on: in both phylogenetic topologies (Figs 1–2), all species from Europe (except *A*. *huijsmanii*) mix with species from Tibetan plateau and form the clade I under fully support at terminal position of the trees, which is originating about 13 Ma. *Agaricus huijsmanii* which is the only Europe species out of clade I, furthermore it is isolated from all other clades with a quite older divergence time of 22 Ma. Based on those results we conclude that most of the presently Europe species of section *Minores* transmigrate from South Asia, through Tibet plateau, then reach Europe in the middle Miocene. Their descendants are represented by species of clade I with age of 13 Ma in this study. However before this time, there are some pioneer species from out of South Asia reach Europe directly in the early Miocene (ca. 22 Ma), but their descendants are quite limited and *A*. *huijsmanii* is the only known species now.

The third route is for species of section *Minores* from tropical Asia spread towards north, reach Northeast Asia around 9 Ma and through Alaska to the West America, their descendants reflect the species of clade VII (Figs 2–3).

The fourth route revealed from our study is species of section *Minores* from South Asia spread towards south and finally reach Oceania. There are four sequestrate (secotioid) species of section *Minores* that have been discovered^44–46^. In our analysis they are distributed in four clades (VIII, XII, XIII and XV) and in three different divergence times. Then we concluded there are at least three invading events occurred through this route that species section *Minores* from tropic Asia dispersed to Oceania: first time is in 16–18 Ma, the species now nest in Clades XV; second time is around 12 Ma and represented by clades XII and XIII; and the most recent time is around 9 Ma represented by clade VIII.

The pattern of the dispersal routes revealed from this study are generally identical with ectomycorrhizal mushrooms^26^, wood-decaying mushrooms^47^, such as tropic origin, dispersed towards west, then reach Europe; towards north, reach Northeast Asia, then through Alaska to West America; towards south and reach Oceania. Moreover, our study suggests a new route for section *Minores* species that dispersed from out of South Asia to Europe directly, which different with the well-known route from tropic Asia through Tibetan plateau to Europe. Does this new dispersal route exist in saprotrophic mushrooms require further studies.

The ferocious arid climate in central Australia makes most species in section *Minores* evoluted into secotioid species. Four secotioid species can be found in Australia: *Agaricus colpeteii* T. Lebel, *A*. *lamelliperditus* T. Lebel & M.D. Barrett, *A*. *wariatodes* (Grgur.) T. Lebel and *A*. *chartaceus* T. Lebel^42, 44, 45^, and they are all located in four different phylogenetic clades with different divergence times. Different phylogenetic clades and divergence times represented different invading events. Our study indicates at least three invading events occurred from tropic Asia to Oceania. This is the reason why four of the five secotioid *Agaricus* species occur in Australia, and all cluster in different phylogenetic clades.

#### Taxonomy

Combined with the phylogenetic analysis and morphological characteristics, sixteen species new to science and three new record species from China are introduced. The diagnosis and morphological features of every species described in this study are summarized in Table 2.

**Figure 4:**
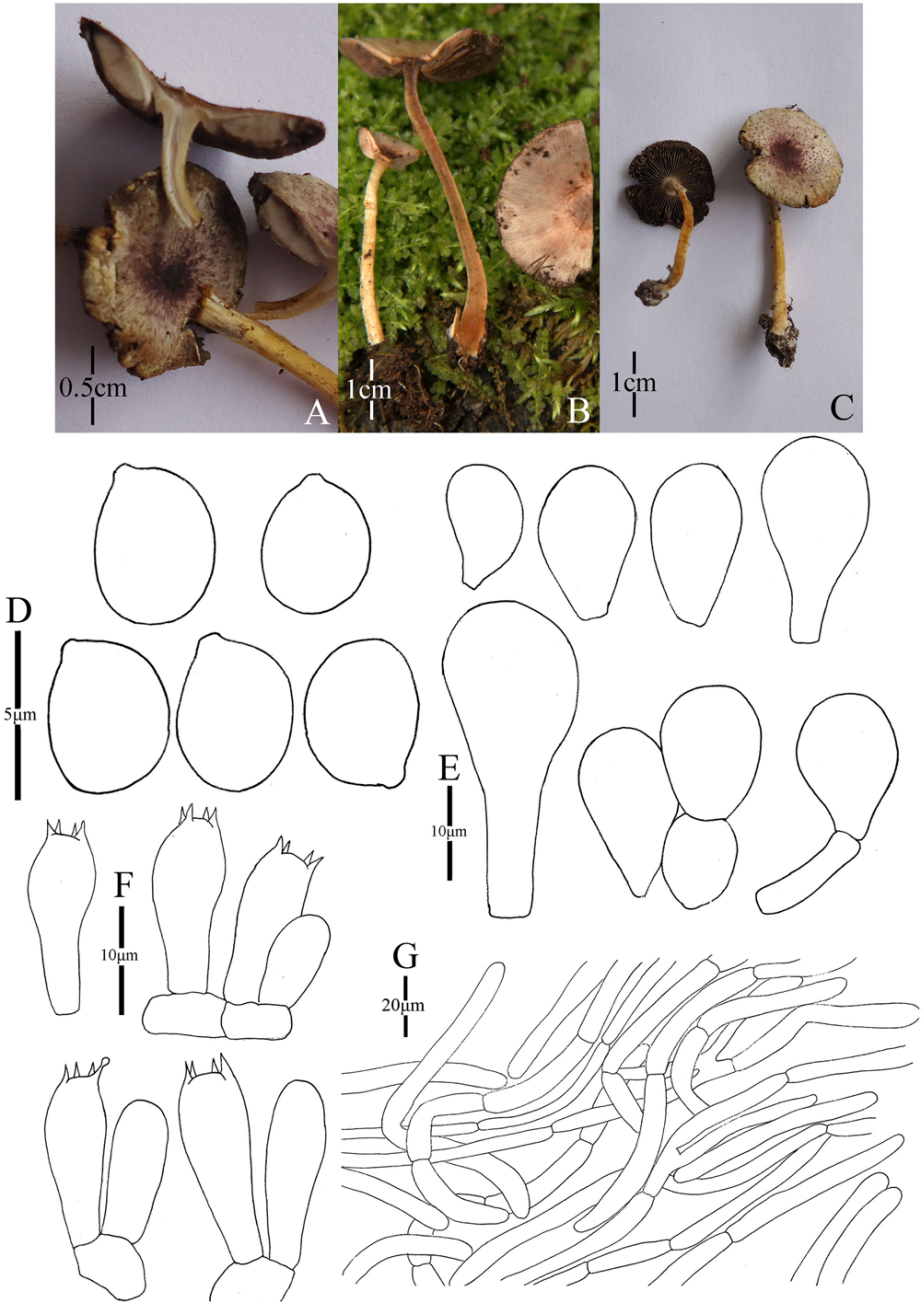
Morphology of *Agaricus blatteus* (*ZRL2012004*, holotype), (**A**–**C**): Basidiome in field (**A**,**C**: *ZRL2012004* (**B**): *ZRL2014282*); (**D**): Basidiospores, (**E**): Cheilocystidia, (**F**): Basidia, and (**G**): Pileipellis hyphae.

1. *Agaricus blatteus* M.Q. He & R.L. Zhao *sp*. *nov*.; Fig. 4.

Fungal Names: FN570357

Faceoffungi Number: FoF 02921

*Etymology*: the epithet “*blatteus*” means dark purple, refers to the dark purple scales on the cap.

*Holotype*: Jindian Forest Park, Kunming, Yunnan Prov., China, 28 June 2012, collected by Zhao Rui-Lin, *ZRL2012004* (HMAS278043, holotype).

Original description: *Pileus* 13–28 mm in diam., plane, disc subumbonate; margin straight, slightly uplifted when mature; surface dry, covered by fibrils completely, and form fibrillose scales, triangular, appressed, denser at disc, scanty towards the margin, dark purple, purple brown; fibrils often rubbed by rain drops; background white or light gray. *Context* up to 1 mm thick, flesh, white. *Lamellae* 2 mm broad, far free, crowded, broad, light brown, dark brown in age, edge even. *Annulus* 1–2 mm in diam., fragile, membranous, single, white, pendant. Smooth at the both sides. *Stipe* 25–51 × 1–6 mm, white, cylindrical, hollow; surface dry, smooth. Odour of almonds. Basidiome flavescent when touching, bruising and cutting.

KOH reaction: positive yellow; Schäffer’s reaction: positive, reddish orange on dry specimen.

*Basidiospores* 4.1–4.9 × 3.0–3.5 μm, [x = 4.5 ± 0.2 × 3.3 ± 0.1, Q = 1.2–1.5, Q_m_ = 1.4 ± 0.1, n = 20], ellipsoid, smooth, thick-walled, brown. *Basidia* 13.4–17.3 × 5.3–7.0 μm, clavate, hyaline, 4-spored, smooth. *Cheilocystidia* 12–33.3 × 6.6–14 μm, smooth, mostly pyriform and broad clavate, some ellipsoid, with yellow pigment inside. If the terminal element appears, cylindrical most, rarely clavate. *Pleurocystidia* absent. *Pileipellis* a cutis composed of hyphae of 3.3–11.5 μm in diam., smooth, cylindrical, light brown, slightly constricted at septa.

*Habitat*: solitary on soil in forest.

*Other specimens examined*: Gantong Temple, Dali, Yunnan Prov., China, 1st August 2014, collected by He Mao-Qiang, *ZRL2014282* (HMAS275774).

Notes: *Agaricus blatteus* is characterized by its small basidiome and dark purple fibrils on the cap. In the phylogeny (Figs 1–2), *A*. *blatteus* represented by specimen *ZRL2012004* is sister to another new species *A*. *dilatostipes* and forms a clade under the supports of 1.0/91 PP/BS values. But in the morphology, *A*. *blatteus* is obviously different from *A*. *dilatostipes* by its small basidiome which of *A*. *dilatostipes* is lager sized (44–110 mm diam. in cap). Combined this new species with all known species of section *Minores* in morphology, *A*. *purpurellus* F.H. Møller and *A*. *parvibicolor* L.J. Chen, R.L. Zhao & K.D. Hyde are two most similar species because they all have purplish fibrils on pileus, and the same basidiospores in size and shape. However, *A*. *purpurellus* has a middle-sized basidiome (pileus 20–50 mm in diam.)^4^, which *A*. *blatteus* has much smaller basidiome (pileus 13–28 mm in diam.). Cheilocystidia of *A*. *parvibicolor* are capitate with long narrow stipe, while those of *A*. *blatteus* are pyriform and broad clavate^19^. Furthermore, the phylogenetic analysis also indicates this new species is different species from all known species.

**Figure 5:**
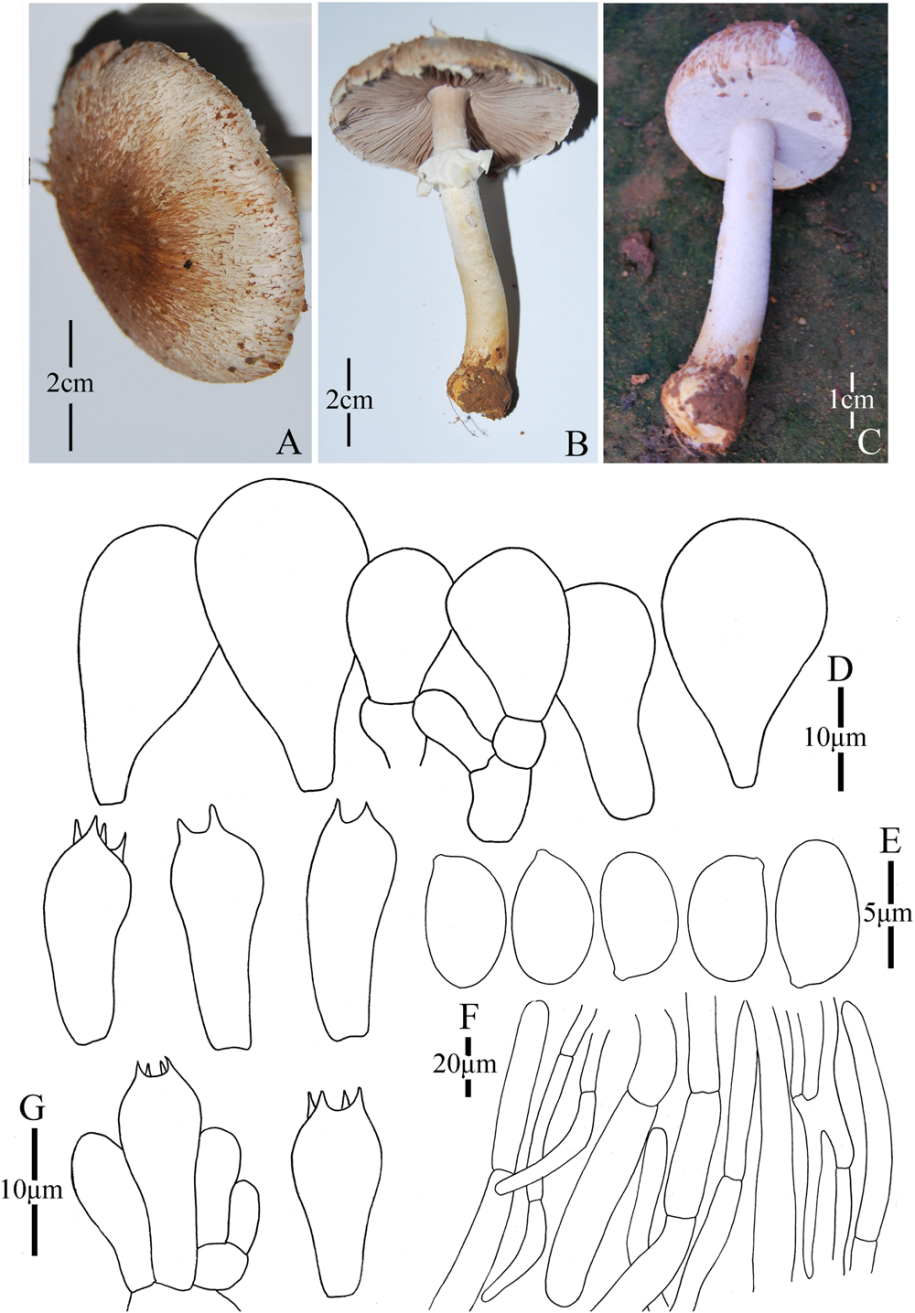
Morphology of *Agaricus bonussquamulosus* (*ZRL2010106*, holotype), (**A**–**C**): Basidiome in field (*ZRL2010106*), (**D**). Cheilocystidia, (**E**). Basidiospores, (**F**). Pileipellis hyphae, and (**G**): Basidia.

2. *Agaricus bonussquamulosus* M.Q. He & R.L. Zhao *sp*. *nov*.; Fig. 5.

Fungal Names: FN570359

Faceoffungi Number: FoF 02922

*Etymology*: the epithet “*bonus*” means well developed, “*squamulosus*” means squamules, “*bonussquamulosus*” refers to the distinct squamules on the surface of pileus.

*Holotype*: Xiaomengyang, Xishangbanna, Yunnan Prov., China, 26 July 2010, collected by Zhao Rui-Lin, *ZRL2010106* (HMAS275803, holotype).

Original description: *Pileus* 55 mm in diam., parabolic when young, then convex; disc slightly depressed; margin straight with appendiculate remains of universal veil; surface dry, completely covered by fine fibrils, and forms fibrillose scales, brown, mess, appressed, denser at disc, scattered towards the margin radially; background white. *Context* 6 mm thick, flesh, white. *Lamellae* 6 mm broad, free, crowded, pink, pinkish-brown, brown in age, edge white, even, intercalated with lamellulae. *Annulus* 8 mm in diam., membranous, single, white, pendant, smooth at both sides. *Stipe* 70 × 8 (15 at base) mm, white, cylindrical with bulbous base, narrow hollow; surface dry, fibrillose, white. Odour of strongly almonds. Basidiome strongly flavescent when touching, bruising or cutting (especially at the base of the stipe).

KOH reaction: positive yellow; Schäffer’s reaction: positive, reddish orange on dry specimen.

*Basidiospores* 5.4–6.4 × 3. 0–4.0 μm, [x = 5.8 ± 0.3 × 3.5 ± 0.2, Q = 1.5–1.8, Q_m_ = 1.7 ± 0.1, n = 20], ellipsoid, elongate, smooth, thick-walled, brown. *Basidia* 12.1–18 × 5.5–7.5 μm, clavate, hyaline, 4-spored, smooth. *Cheilocystidia* 13.5–30.5 × 7.6–18 μm, smooth, pyriform most, some broadly clavate, hyaline. *Pleurocystidia* absent. *Pileipellis* a cutis composed of hyphae of 4.8–14.0 μm in diam., smooth, cylindrical, hyaline, light yellow, slightly constricted at septa.

*Habitat*: solitary on soil in forest.

Notes: This new species is characterized by its heavily brown fibrils on the surface of pileus and pyriform cheilocystidia. This new species nests with *A*. *robustulus* L.J. Chen, Callac, L.A. Parra, K.D. Hyde & De Kesel, *A*. *fimbrimarginatus* L.J. Chen, Callac & K.D. Hyde and *A*. sp./CA846 under the support of 1.0/88 PP/BS values, and presents as Clade XIV in this study (Figs 1–2). In the morphology, this new species is different from *A*. *robustulus* by its heavily fibrils on the surface of pileus, while those of *A*. *robustulus* is forming triangular scales; *A*. *fimbrimarginatus* has purplish fibrils on the cap, and those of *A*. *bonussquamulosus* is brown colored, also, the relative larger basidiospores of *A*. *fimbrimariginatus* (4.7 ± 0.11 × 3.2 ± 0.09) is another difference between these two species^10^.

**Figure 6:**
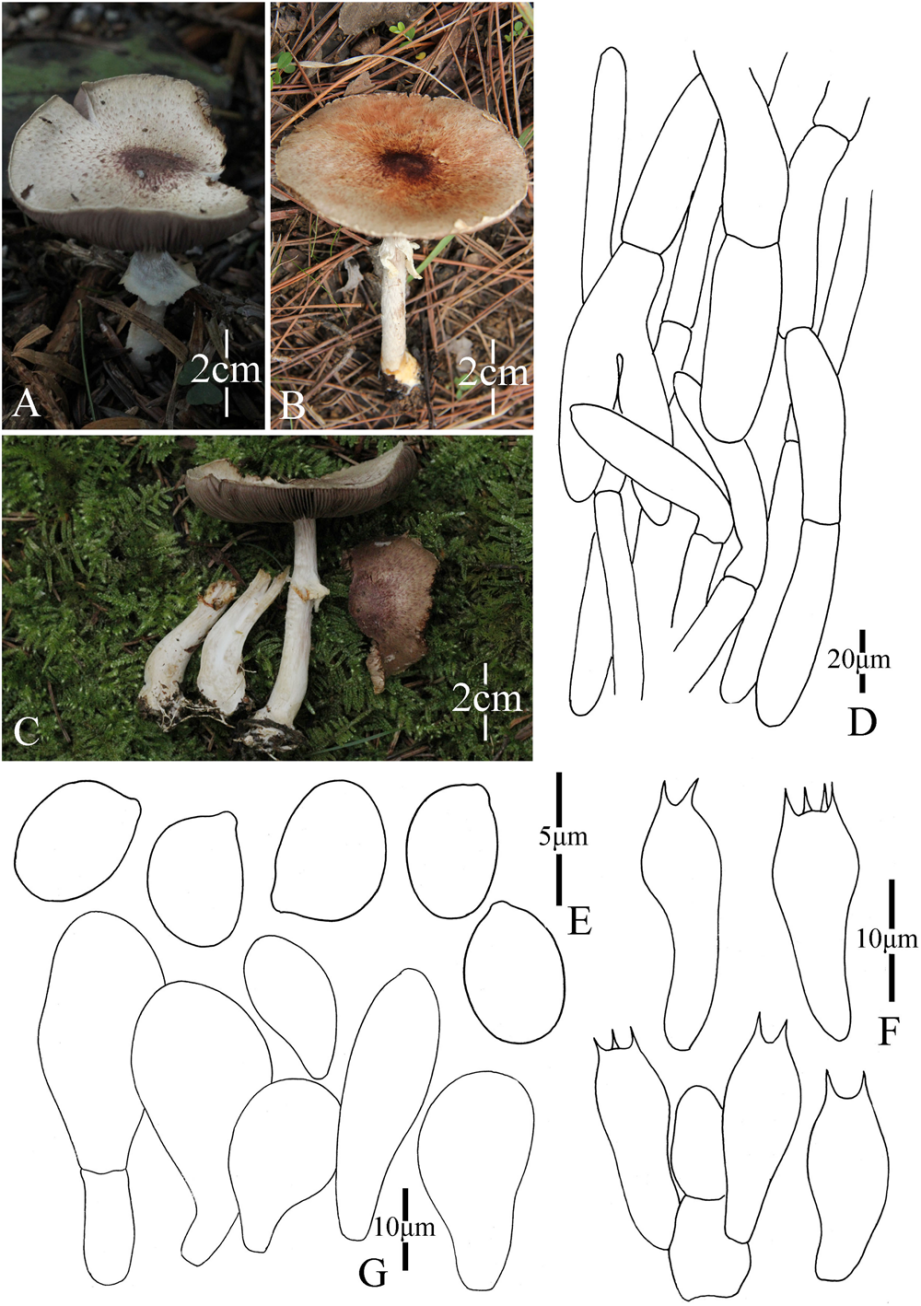
Morphology of *Agaricus jingningensis* (*ZRL20151562*, holotype), (**A**–**C**): Basidiome in field (**A**, **C**). *ZRL20151562*; (**B**). *ZRL2014248*); (**D**). Pileipellis hyphae; (**E**). Basidiospores; (**F**). Basidia, and (**G**). Cheilocystidia.

3. *Agaricus jingningensis* M.Q. He & R.L. Zhao *sp*. *nov*.; Fig. 6

Fungal Names: FN570356

Faceoffungi Number: FoF 02923

*Etymology*: the epithet “*jingning*” refers to the location name of the holotype.

*Holotype*: Jingning County, Lishui, Zhejiang Prov., China, 19 August 2015, collected by Ling Zhi-lin, *ZRL20151562* (HMAS275787, holotype).

Original description: *Pileus* 32–78 mm in diam., convex first, plane with age; disc umbonate, margin straight, also can be uplifted in age, margin slightly exceeding; surface dry, covered by plenty of fibrils at whole cap, fibrils can be rubbed by raindrop; background white or gray, get red in wet; fibrillose scales reddish brown or purplish brown, denser at disc, scanty obviously towards the margin; tiny fibrillose scales triangular-shaped, appressed, or erected. *Context* 3–4 mm thick, flesh, white or light gray. *Lamellae* 4–5 mm broad, free, crowded, edge even, pinkish brown to brown in age, intercalated with lamellulae. *Annulus* up to 10 mm in diam., fragile, membranous, single, white, pendant, smooth on both sides. *Stip*e 60 × 5–7 (10–11 at base) mm, white, hollow, cylindrical, slightly bulbous at base, surface dry, with white fibrils below the annulus. Odour of almonds. Basidiome flavescent when touching and bruising, then becoming orange brown after several minutes. No discoloration or slightly yellowish on cutting.

KOH reaction: positive yellow; Schäffer’s reaction: positive, reddish orange on dry specimen.

*Basidiospores* 4.3–5.2 × 3.0–3.6 μm, [x = 4.8 ± 0.3 × 3.3 ± 0.1, Q = 1.3–1.6, Q_m_ = 1.4 ± 0.1, n = 20], ellipsoid, smooth, thick-walled, brown. *Basidia* 12.8–18.4 × 5.5–7.6 μm, clavate, hyaline, 4-spores, smooth. *Cheilocystidia* 14.5–32 × 7–17 μm, smooth, clavate and ellipsoid. Septa at base sometimes, with yellow pigment inside. *Pleurocystidia* absent. *Pileipellis* a cutis composed of hyphae of 5.5–14 μm in diam., smooth, cylindrical most, brown, terminal element ellipsoid, constricted at some septa.

*Habitat*: solitary on soil in forest.

*Other specimens examined*: Yingjiang County, Yunnan Prov., China, 21 July 2013, collected by Yu Qing-Hua, *ZRL2013405* (HMAS275752); Shigu, Lijiang, Yunnan Prov., China, 31 July 2014, collected by Su Sheng-Yu, *ZRL2014248* (HMAS275775).

Notes: In our phylogeny analysis (Figs 1–2), the proposed new species clusters with *A*. *catenatus* (another new species from this study) in clades IX under the support of 1.0 PP value. Compared with *A*. *catenatus*, they are sharing the same sized basidiome, similar basidiospores in shape and size, however the cheilocystidia of *A*. *catenatus* are various in shape and often in chains, and those of *A*. *jingningensis* is simple. In the field, they can also be easily separated from *A*. *catenatus* having a nearly white pileus. *Agaricus pallens* (J.E. Lange) L.A. Parra is another species which resembles this new species, because both of them have relative slender basidiome, similar characters in pileus and basidiospores. However, the cheilocystidia of *A*. *pallens* is septa at base or multiseptate mostly, which is different from *A*. *jingningensis* ^4^. Then we proposed *A*. *jingningensis* as a new species, and is characterized by its reddish brown fibril scales at the disc of cap, slender basidiome and simple cheilocystidia.

**Figure 7:**
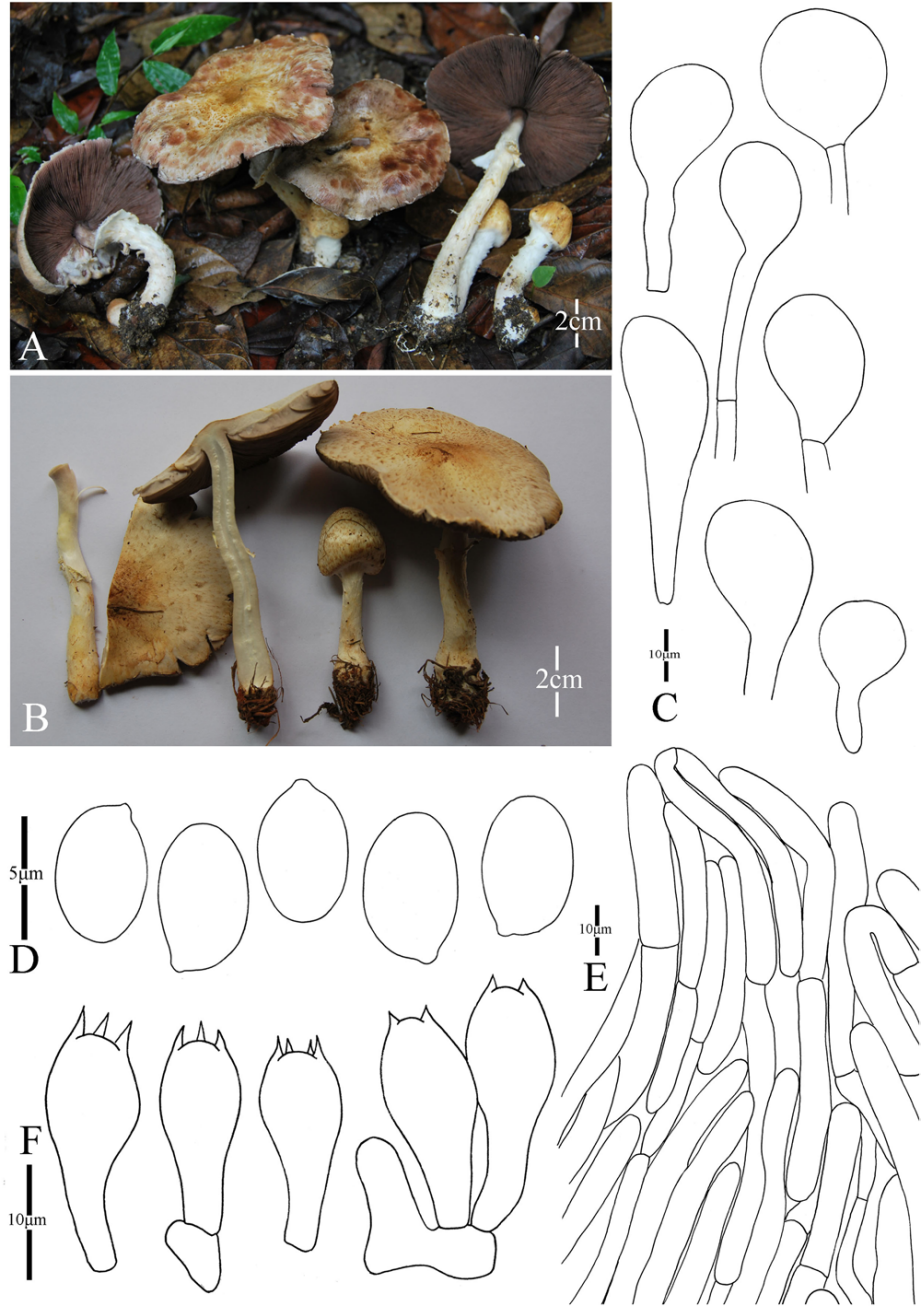
Morphology of *Agaricus cerinupileus* (*ZRL2012001*, holotype), (**A**–**B**): Basidiome in field (**A**: *ZRL2011157*; (**B**): *ZRL2012001*), (**C**): Cheilocystidia, (**D**): Basidiospores, (**E**): Pileipellis hyphae, and (**F**): Basidia.

4. *Agaricus cerinupileus* M.Q. He & R.L. Zhao *sp*. *nov*.; Fig. 7

Fungal Names: FN570358

Faceoffungi Number: FoF 02924

*Etymology*: the epithet “*cerinu”* means ochraceous-yellow, refers to the pileus colour.

*Holotype*: Southwest Forestry University, Kunming, Yunnan Prov., China, 22 Jun 2012, collected by Zhao Rui-Lin, *ZRL2012001* (HMAS280106, holotype)

Original description: *Pileus* 70–90 mm in diam., parabolic with flat top when young, then applano-convex, finally plane with umbo, margin eroded mostly, or straight, margin exceeding with white appendiculate elements of universal veil; surface dry covered by fibrils at whole cap; background white or light yellow, turn red in wet; fibrillose scales ochraceous-yellow, triangular, appressed, denser on disc, radially scattered towards the margin. *Context* 2–8 mm thick, flesh, white, brown in old. *Lamellae* 3–7 mm broad, free, crowded, pink or pinkish brown firstly, then brown, edge even, normal to ventricose, intercalated with lamellulae. *Annulus* 8–15 mm in diam., single, membranous, pendant, smooth on both sides, white, turn yellow when dry or old. *Stipe* 47–128 × 6–65 (11–90 at base) mm, white or ochraceous-yellow, hollow, long clavate, surface dry, above the annulus smooth, below heavily fibrillose-woolly especially when young. Odour of almonds. Basidiome flavescent when touching, bruising and cutting.

KOH reaction: positive yellow; Schäffer’s reaction: positive, reddish orange on dry specimen.

*Basidiospores* 5.5–6.5 × 3.5–4.2 μm, [x = 6.0 ± 0.3 × 3.5 ± 0.2, Q = 1.4–1.7, Q_m_ = 1.6 ± 0.1, n = 20], ellipsoid, elongate, smooth, thick-walled, brown. *Basidia* 13.4–22 × 5.4–7 μm, clavate, hyaline, 4-spored, smooth. *Cheilocystidia* 18.6–44 (−48) × 11–25 μm, smooth, capitate with long narrow stipe mostly, or globose, clavate, broadly clavate, hyaline or containing yellow pigments. *Pleurocystidia* absent. *Pileipellis* a cutis composed of hyphae of 3.3–7.4 μm in diam., smooth, cylindrical, brown, slightly constricted at septa.

Habitat: solitary on soil in forest.

*Other specimens examined*: Gaoligongshan, Baoshan, Yunnan Prov., China, 21 July 2011, collected by Zhao Rui-Lin, *ZRL2011157* (HMAS280107); China, Yunnan Prov., Kunming, Kunming Institute of Botany, 15 Sep 2012, collected by Zhou Jun-Liang, *ZRL2012725* (HMAS275757).

Notes: In our phylogeny analysis (Figs 1–2), the proposed new species *A*. *cerinupileus* was represented by four specimens, and they cluster together under the support of 1.0/100 PP/BS values in the clade XI. The phylogenic closest species is *A*. *luteopallidus* L.J. Chen, Karunarathna, R.L. Zhao & K.D. Hyde^10^. In morphology, the stipe surface of *A*. *luteopallidus* is slightly fibrillose, while *A*. *cerinupileus* is heavily fibrillose especially when young. There are also several species with yellowish brown caps, such as *A*. *luteofibrillosus* M.Q. He, L.J. Chen & R.L. Zhao, *A*. *luteoflocculosus* Kalaméés and *A*. *fulvoaurantiacus* L.J. Chen & Karunarathna^4, 8, 10^. *Agaricus cerinupileus* is a different species from *A*. *luteofibrillosus* and *A*. *fulvoaurantiacus* because the later two species nest in the Clade III based on the phylogeny (Figs 1–2). In morphology, *A*. *cerinupileus* differs from *A*. *fulvoaurantiacus* by its heavily fibrils on the stipe surface below the annulus, while those of *A*. *fulvoaurantiacus* is weak^10^; differs from *A*. *luteoflocculosus* by its capitate cheilocystidia and larger basidiospores, while those of *A*. *luteoflocculosus* are simple cheilocystidia and basidiospores 5.1–5.3 × 3.4–3.7 μm^4^. In summary, this new species is characterized by its pileus orchraceous-yellow, cheilocystidia capitate with long narrow stipe and containing yellow pigments.

**Figure 8:**
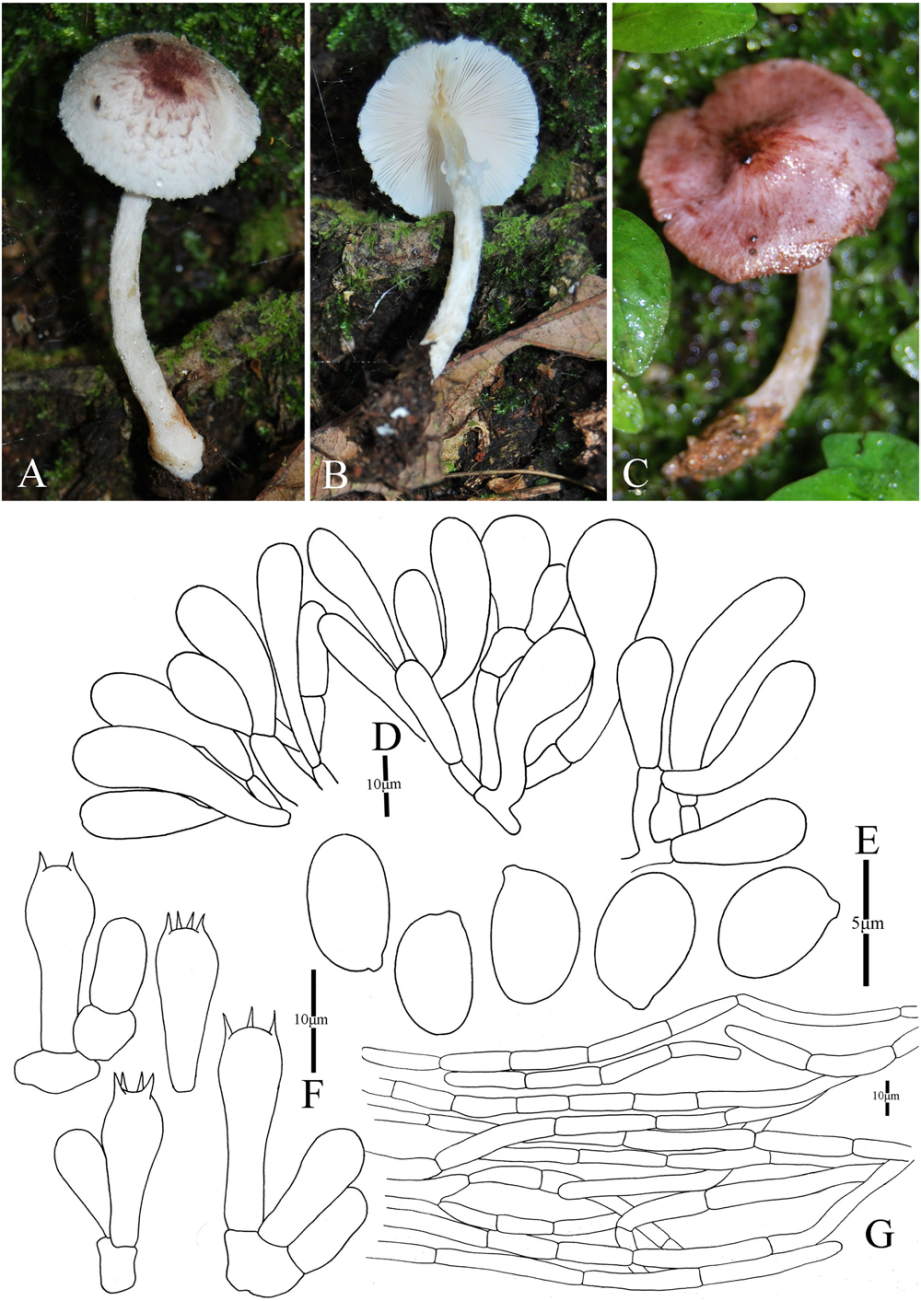
Morphology of *Agaricus minorpurpureus* (*ZRL2010058*, holotype), (**A**–**C**): Basidiome in field (**A**,**B**; *ZRL2010058*; (**C**): *ZRL2013342*), (**D**): Cheilocystidia, (**E**): Basidiospores, (**F**): Basidia, and (**G**): Pileipellis hyphae.

5. *Agaricus minorpurpureus* M.Q. He & R.L. Zhao *sp*. *nov*.; Fig. 8

Fungal Names: FN570349

Faceoffungi Number: FoF 02925

*Etymology*: the epithet “*minor*” means small basidiome, and “*purpureus*” means purple fibrils on pileus.

*Holotype*: Manda village, Mangao, Xishuangbanna, Yunnan Prov., China, 24 June 2010, collected by Zhao Rui-Lin *ZRL2010058* (HMAS275776, holotype).

Original description: *Pileus* 16–18 mm in diam., plane, convex; disc slightly unbonate; margin straight, exceeding; surface dry, covered by fibrils at the whole cap; background white, turn into purple red in wet; fibrils reddish brown or purplish brown, forming fibrillose scales at disc, and fading towards the margin. *Context* 1 mm thick, flesh, white. *Lamellae* 2–3 mm broad, far free, crowded, pink or pinkish brown first, then brown in age, edge even, ventricose, intercalated with lamellulae. *Annulus* 2–3 mm in diam., single, fragile, membranous, pendant, white. *Stipe* 12–40 × 2 (4–5 at base) mm, white, hollow, cylindrical, bulbous at base with rhizomorphs, surface dry, silky, fibrillose below the annulus. Odour of almonds. Basidiome flavescent when touching, no discoloration on cutting.

KOH reaction: positive yellow; Schäffer’s reaction: positive, reddish orange on dry specimen.

*Basidiospores* 4.7–5.3 × 2.9–3.7 μm, [x = 5.0 ± 0.2 × 3.3 ± 0.2, Q = 1.4–1.6, Q_m_ = 1.5 ± 0.1, n = 20], ellipsoid, smooth, thick-walled, brown. *Basidia* 13.3–19.5 × 5.3–6.9 μm, clavate, hyaline, 4-spored, smooth. *Cheilocystidia* 21–45 × 6–11.6 μm, abundant, clavate, smooth, hyaline with yellow pigments inside. *Pleurocystidia* absent. *Pileipellis* a cutis composed of hyphae of 2.2–7.6 μm in diam., smooth, cylindrical, brown, slightly constricted at septa.

*Habitat*: solitary on soil in forest.

*Other specimens examined*: Tongbiguang, Yingjiang County, Yunnan Prov., China, 20 July 2013, collected by He Mao-Qiang *ZRL2013342* (HMAS275759).

Notes: In our phylogenetic analysis, *A*. *minorpurpureus* are represented by specimens *ZRL2010058* and *ZRL2013342*. This two specimens form a distinct clade with the support of 1.0/100 PP/BS values in clade XIII (Figs 1–2). *Agaricus minorpurpureus* is a species with tiny basidiome (pileus diam. less than 20 mm), and several species of section *Minores* has such small basidiome, such as *A*. *callacii* L.A. Parra, R. Iglesias, Fern.-Vic. & Oyarzabal and *A*. *parvibicolor*. But *A*. *callacii* has larger basidiospores (5.7–6.7 × 4.2–5.36 μm) and dark brown scales on the pileus^4^, which are different from those of *A*. *minorpurpureus*. Also, only present clavate cheilocystidia makes *A*. *minorpurpureus* differ from *A*. *parvibicolor* and *A*. *gemloides* M.Q. He & R.L. Zhao^5^. The cheilocystidia of *A*. *parvibicolor* and *A*. *gemloides* are pyriform, globose or capitate with long stipe^19^. Considering the distinct morphological features, such as its extremely small basidiome, purplish brown scales, abundant of clavate cheilocystidia, and distinct phylogenetic position of *A*. *minorpurpureus*, we proposed it is new to science.

**Figure 9:**
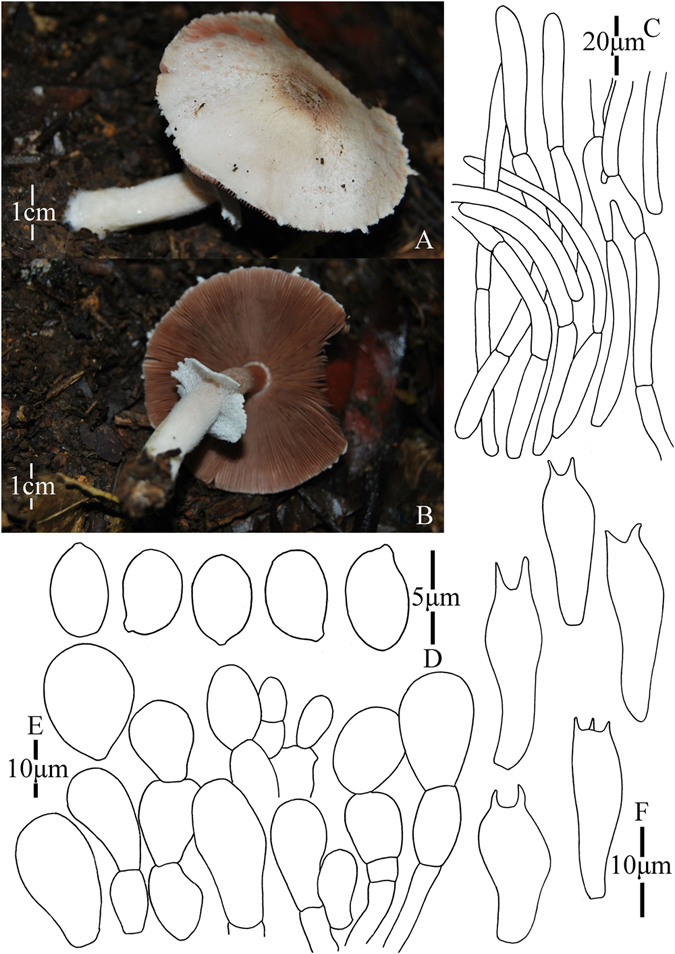
Morphology of *Agaricus catenatus* (*ZRL2012104*, holotype), (**A**,**B**): Basidiome in field (*ZRL2012104*), (**C**): Pileipellis hyphae, (**D**): Basidiospores, (**E**): Cheilocystidia, and (**F**): Basidia.

6. *Agaricus catenatus* M.Q. He & R.L. Zhao *sp*. *nov*.; Fig. 9

Fungal Names: FN570355

Faceoffungi Number: FoF 02926

*Etymology*: the epithet “*catenatus*” means chains, refers to the cheilocystidia are in chains.

*Holotype*: Nuozhang Village, Cangyuan County, Yunnan Prov., China, 6 July 2012, collected by Zhao Rui-Lin, *ZRL2012104* (HMAS275760, holotype)

Original description: *Pileus* 50 mm in diam., plane; disc slightly truncated; margin straight with white appendiculate remains of universal veil; surface dry, fibrillose, appressed, white generally excepting light brown to reddish brown at disc; background white or light gray, turning red in wet. *Context* 3 mm thick at disc, flesh, white. *Lamellae* up to 3 mm broad, far free, crowded, pink brown to brown. *Annulus* single, fragile, membranous, white, pendant, smooth on both sides. *Stipe* 55 × 6 mm, hollow, white, cylindrical; surface dry, smooth above the annulus, fibrillose below the annulus. Odour of almonds. Basidiome no discoloration or slightly flavescent when touching. Discoloration yellowish brown after several minutes on cutting.

KOH reaction: positive yellow. Schäffer’s reaction: positive, reddish orange on dry specimen.

*Basidiospores* 4.6–5.4 × 2.7–3.5 μm, [x = 5.1 ± 0.2 × 2.7 ± 0.1, Q = 1.4–1.8, Q_m_ = 1.4 ± 0.1, n = 20], ellipsoid, elongate, smooth, thick-walled, brown. *Basidia* 14.5–20.5 × 4.5–7.7 μm, long clavate, hyaline, 4-spored, smooth. *Cheilocystidia* 13.3–33.7 × 10.8–18.3 μm, mostly catenulate by 2–3 elements of clavate, globose or ellipsoid, smooth, hyaline, some with yellow pigments inside. *Pleurocystidia* absent. *Pileipellis* a cutis composed of hyphae of 3.3–11 μm in diam., smooth, cylindrical, yellowish brown, no constricted at septa.

*Habitat*: solitary in forest.

Notes: In phylogeny analysis, this new species is sister to *A*. *jingningensis* under the support of 0.9/- PP/BS values in clade IX (Figs 1–2). The morphological difference between them are presented in *A*. *jingningensis* part. Generally this new species is characterized by its nearly white pileus and the catenulate cheilocystidia, and the latter is rare for the species of section *Minores*.

**Figure 10:**
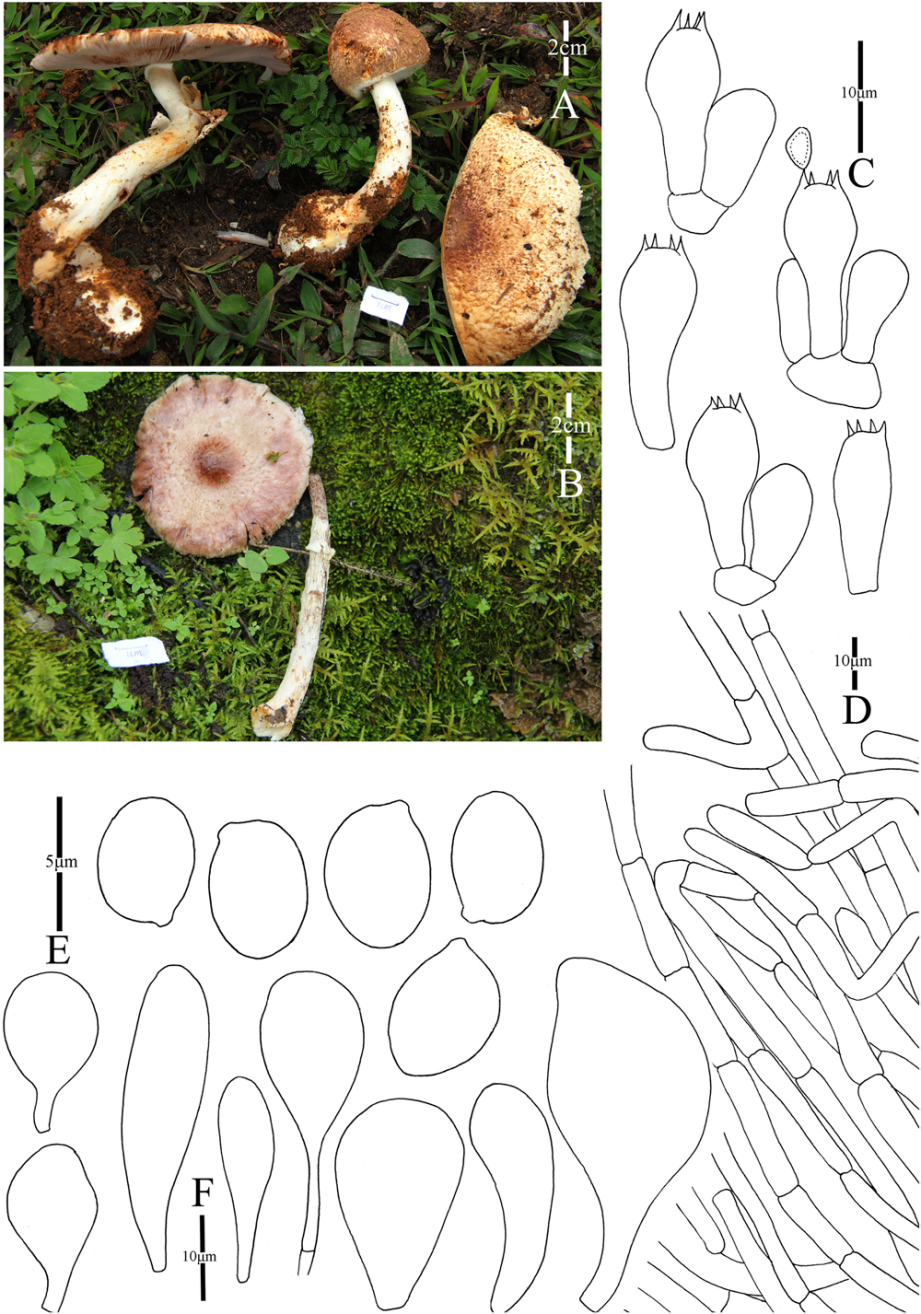
Morphology of *Agaricus dilatostipes* (*ZRL2014421*, holotype), (**A**,**B**): Basidiome in field (**A**: *ZRL2014421*; (**B**): *ZRL2014450*), (**C**): Basidia, (**D**): Pileipellis hyphae, (**E**): Basidiospores, and (**F**): Cheilocystidia.

7. *Agaricus dilatostipes* M.Q. He & R.L. Zhao *sp*. *nov*., Fig. 10

Fungal Names: FN570354

Faceoffungi Number: FoF 02927

*Etymology*: the epithet “*dilato*” means inflated, and “*dilatostipes”* refers to the stipe is inflated at the base.

*Holotype*: Tacheng, Weixi County, Yunnan Prov., China, 5 August 2014, collected by Su Sheng-Yu, *ZRL2014421* (HMAS254647, holotype).

Original description: *Pileus* 44–110 mm in diam., parabolic first, then convex, finally plane; disc slightly unbonate; margin straight, exceeding; surface dry, with plenty of fibrils covered whole cap; background white or light brown, turn into red when water-soaked; fibrillose scales reddish brown, triangular, appressed, denser on disc, scanty towards the margin. *Context* 4–6 mm thick, flesh, white. *Lamellae* 4–6 mm broad, far free, crowded, pink first, then brown, edge even, intercalated with lamellulae. *Annulus* 10–30 mm in diam., single, white, fragile, membranous, pendant, smooth on both sides. *Stipe* 90–150 × 4–10 mm (6–30 mm at base), white, hollow, cylindrical, bulbous at base, surface dry, silky, slightly fibrillose. Odor of almonds. Basidiome flavescent first when touching and bruising, then turn into orange after few minutes. Yellowish on cutting.

KOH reaction: positive yellow. Schäffer’s reaction: positive, reddish orange on dry specimen.

*Basidiospores* 4.5–5.6 × 3.0–3.5 μm, [x = 5.1 ± 0.3 × 3.3 ± 0.2, Q = 1.4–1.8, Q_m_ = 1.6 ± 0.1, n = 20], ellipsoid, elongate, smooth, thick-walled, brown. *Basidia* 13.8–25.8 × 5.1–7.6 μm, clavate, hyaline, 4-spored, smooth. *Cheilocystidia* 15–40 (−65) × 9.0–20 μm, smooth, simple, clavate most, can be broadly clavate and capitate with long narrow stipe, hyaline. *Pleurocystidia* absent. *Pileipellis* a cutis composed of hyphae of 3.5–9.9 μm in diam., smooth, cylindrical, brown, slightly constricted at septa.

*Habitat*: solitary or scattered on soil in forest.

*Other specimens examined*: Tacheng, Weixi County, Yunnan Prov., China, collected by Xu Meng-Lin, *ZRL2014450* (HMAS275758). Dazhongshan Forestry Factory, Nanhua, Yunnan Prov., China, 11 September 2015, collected by Zhou-Junliang, Z*RL20151110* (HMAS275762).

Notes: In phylogeny analysis, *A*. *dilatostipes* is sister to *A*. *blatteus* under the support of 1.0/91 PP/BS values (Figs 1–2). In the morphology, *A*. *blatteus* has a small cap (diam. 13–28 mm), while *A*. *dilatostipes* is middle to large sized in pileus (pileus 44–110 mm diam.). Another distinct morphological character of *A*. *dilatostipes* is its bulbous base at the stipe, which is similar to those of *A*. *dulcidulus* Schulzer, *A*. *jacobi* L.A. Parra, A. Caball. & Callac, *A*. *matrum* L.A. Parra, A. caball., S. Serrano, E. Fernández & Callac and *A*. *purpurellus* ^4^. However, the basidiospores of *A*. *dulcidulus* is smaller (3.6–4.8 × 2.6–3.3 μm) than those of *A*. *dilatostipes*; the cheilocystidia of *A*. *jacobi* is multiseptate, which is different from *A*. *dilatostipes*; *A*. *matrum* and *A*. *purpurellus* have much smaller basidiome (pileus 15–50 mm in diam.) than those of *A*. *dilatostipes* ^4^. Then we proposed *A*. *dilatostipes* as a new species. This new species is distinguished by its large basidiome, obviously bulbous base of stipe and simple cheilocystidia.

**Figure 11:**
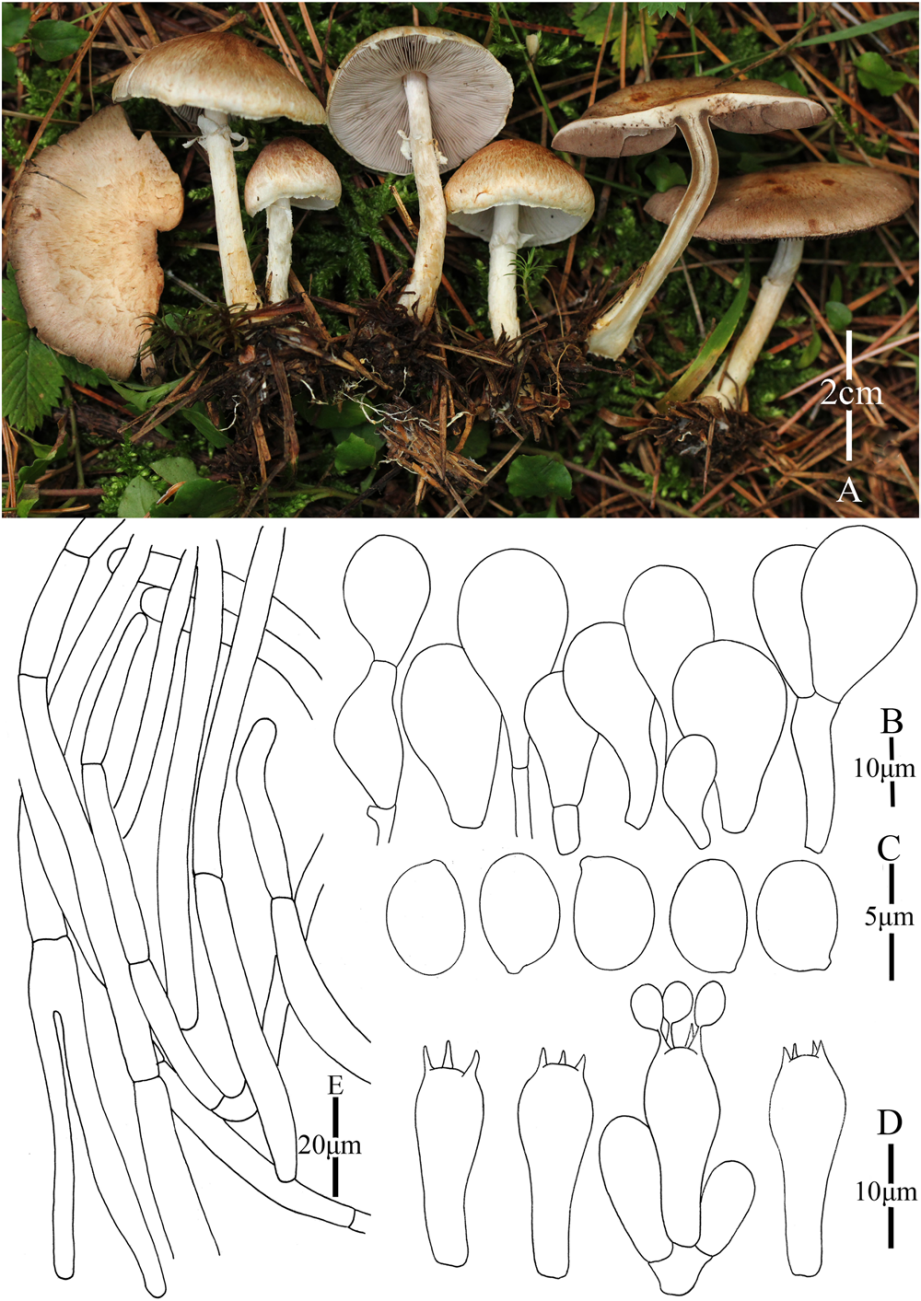
Morphology of *Agaricus armandomyces* (*ZRL2015992*, holotype), (**A**): Basidiome in field (*ZRL2015992*), (**B**): Cheilocystidia, (**C**): Basidiospores, (**D**): Basidia, and (**E**): Pileipellis hyphae.

8. *Agaricus armandomyces* M.Q. He & R.L. Zhao *sp*. *nov*.; Fig. 11

Fungal Names: FN570353

Faceoffungi Number: FoF 02928

*Etymology*: the epithet refers to the habitat of this species a forest of *Pinus armandii*.

*Holotype*: Ludian County, Zhaotong, Yunnan Prov., China, alt. 2000–3000 m, 2 August 2015, collected by Bai Xu-Ming, *ZRL2015992* (HMAS275768, holotype).

Original description: *Pileus* 16–42 mm in diam., convex first, plane with age, disc umbonate, or truncate umbonate, margin decurved when young, then straight with age, slightly exceeding; surface dry, covered by fibrils at the whole cap, forming fibrillose scales, light brown, ochre, or reddish brown, dense at disc and scattered towards the margin; background white or light yellow. *Context* 4 mm thick, flesh, white at pileus and yellowish brown at stipe. *Lamellae* 5–6 mm broad, free, crowded, broad, first white, pink or pinkish brown, then brown, edge even, intercalated with lamellulae. *Annulus* up to 3 mm in diam., fragile, membranous, single, white, pendant, smooth at both sides. *Stipe* 38–40 × 3–5 mm (6–7 mm at base), cylindrical, sometimes with a swallow base, hollow, surface dry, white, fibrillose below the annulus. Odour of almonds. Basidiome flavescent, orange when touching and bruising. Context turning flavescent first, then brown on exposure after few minutes.

KOH reaction: positive yellow. Schäffer’s reaction: positive, reddish orange on dry specimen.

*Basidiospores* 4.7–5.3 × 3.2–3.7 μm, [x = 4.9 ± 0.2 × 3.4 ± 0.1, Q = 1.4–1.6, Q_m_ = 1.4 ± 0.0, n = 20], ellipsoid, smooth, thick-walled, brown. *Basidia* 15.7–21.2 × 6.2–8.0 μm, clavate, hyaline, 4-spored, smooth. *Cheilocystidia* 16.2–40 × 8.2–16.7 μm, smooth, pyriform mostly, broadly clavate and capitate with long narrow stipe, occasionally septate at the base with the cylindrical terminal element, hyaline. *Pleurocystidia* absent. *Pileipellis* a cutis composed of hyphae of 4.2–8.2 μm in diam., smooth, cylindrical, brown, slightly constricted at septa in some cases.

*Habitat*: solitary or scattered on soil in artificial forest which is dominant by *Pinus armandii* Franch.

*Other specimens examined*: Ludian County, Zhaotong, Yunnan Prov., China, alt. 2000–3000 m, 2 August 2015, collected by Zhao Rui-Lin, *ZRL2015999* (HMAS275769); same location, 2 August 2015, collected by Su Sheng-Yu *ZRL2015991* (HMAS275770); same location, 2 August 2015, collected by He Mao-Qiang, *ZRL2015998* (HMAS275771), *ZRL20151023* (HMAS275789), *ZRL2015997* (HMAS275767); Tahe County, Great Hinggan, Heilongjiang Prov., China, 16 August 2015, collected by Li Guo-Jie, *ZRL20151693* (HMAS275785).

Notes: The new species is represented by six specimens from Southwest and Northeast China under the support of 1.0/98 PP/BS values. In phylogeny, the clade of *A*. *armandomyces* is sister to *A*. *kerriganii* L.A. Parra, B. Rodr., A. Caball., M. Martín-Calvo & Callac, *A*. *sp*. (specimen WC912) and *A*. *edmondoi* L.A. Parra, Cappelli & Callac which are origin from Europe and West America in the clade I^4^ (Figs 1–2). In the morphology all of them have discoloration of distinct yellow on touching, similar basidiospores and cheilocystidia in size and shape. Even *A*. *armandomyces* are morphologically similar to the known species *A*. *kerriganii* and *A*. *edmondoi*, they can be molecular identified as different species. For example, there are six informative characters in ITS sequences between *A*. *armandomyces*, *A*. *kerriganii* (KF447893 from type specimen) and *A*. *edmoindoi* (KF447902 from type specimen) respectively (details see Table 3).

**Table 3:** ITS nucleotide difference between *Agaricus armandomyces* and related species.

Samples	Positions in the ITS alignment (713 nts)										
*A*. *armandomyces*	142	496	514	595	624	638	652	653	654	657	662
ZRL2015991	A	G	—	C	T	C	T	—	—	G	G
ZRL2015992	A	G	—	C	T	C	T	—	—	G	G
ZRL2015997	A	G	—	C	T	C	T	—	—	G	G
ZRL2015998	A	G	—	C	T	C	T	—	—	G	G
ZRL20151693	A	G	—	C	T	C	T	—	—	G	G
*A*. *Kerreganii* T	G	G	—	T	T	T	T	T	G	G	A
*A*. *edmondoi* T	A	A	T	T	A	C	C	T	G	A	A

**Figure 12:**
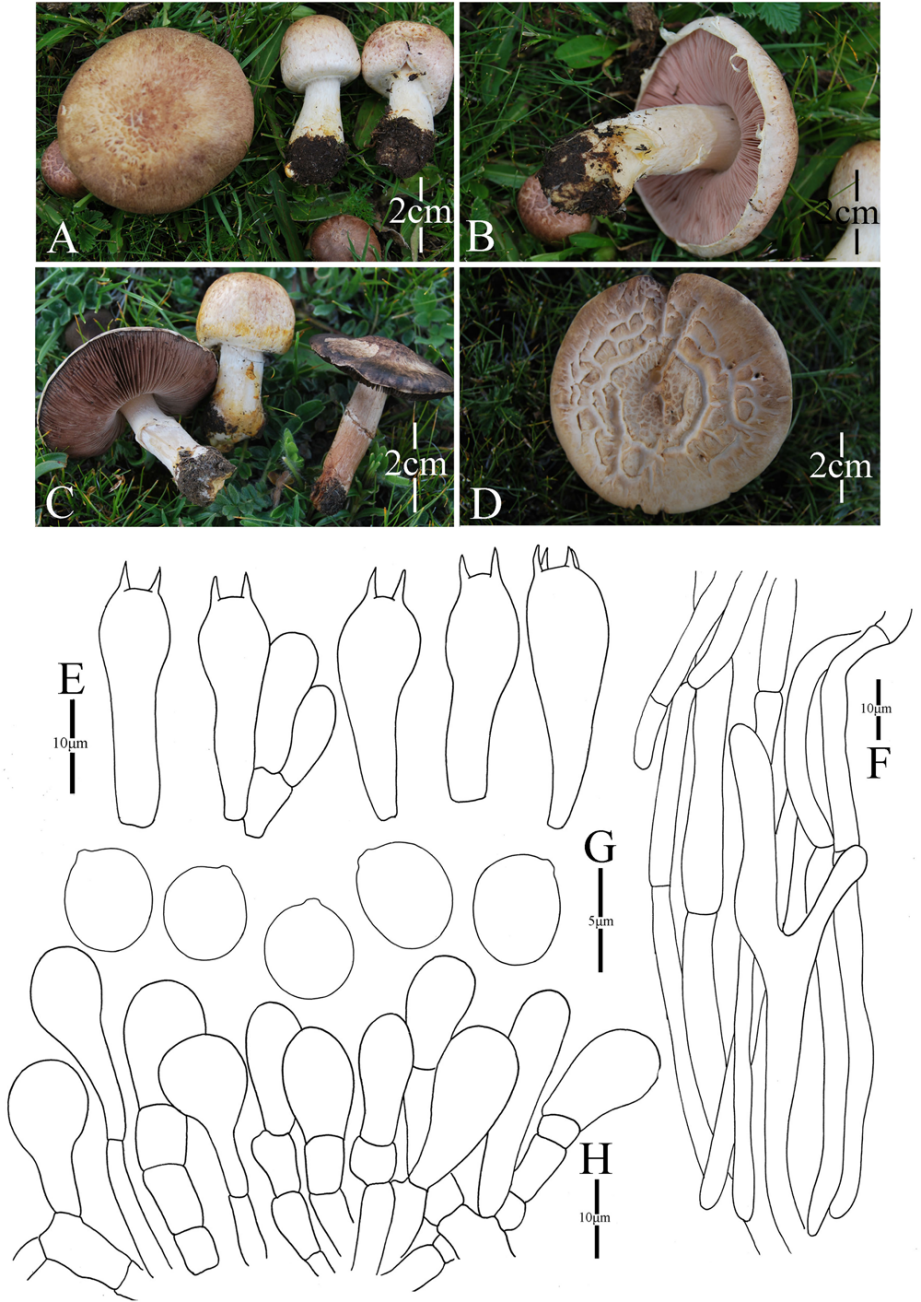
Morphology of *Agaricus globosporus* (*ZRL2012656*, holotype), (**A**–**D**): Basidiome in field (**A**,**B**): *ZRL2012656*, (**C**): *ZRL2012652*, (**D**): *ZRL2012655*), (**E**): Basidia, (**F**): Pileipellis hyphae, (**G**): Basidiospores, and (**H**): Cheilocystidia.

9. *Agaricus globosporus* M.Q. He & R.L. Zhao *sp*. *nov*.; Fig. 12

Fungal Names: FN570351

Faceoffungi Number: FoF 02929

*Etymology*: the epithet “*globo*” means globose, refers to the globose basidiospores.

*Holotype*: Wulashan mountain, Mangkang County, Tibet, China, 5 August 2012, collected by Li Guang-Ping, *ZRL2012656* (HMAS275796, holotype);

Original description: *Pileus* 11–62 mm in diam., parabolic first, then convex, finally plane with age; margin incurved when young, then decurved, finally can be uplifted in age, margin exceeding; surface dry, covered by reddish brown, purple fibrils against white background; fibrils often break into triangular fibrillose scales at disc, appressed; sometimes cracking; turn reddish black in wet. *Context* 5–8 mm thick, flesh, white. *Lamellae* 3–15 mm broad, free, crowded, pink or pinkish brown, then brown, edge even, intercalated with lamellulae. *Annulus* 4–8 mm in diam., single, membranous, white, pendant, smooth on both sides. *Stipe* 22–48 × 6–15 (8–14 at base) mm, white, hollow, clavate when young, then cylindrical, surface dry, slightly fibrous. Odour of almonds. Basidiome strongly flavescent when touching and bruising. Yellowish discoloration on cutting.

KOH reaction: positive yellow. Schäffer’s reaction: positive, reddish orange on dry specimen.

*Basidiospores* 4.2–5.7 × 3.9–4.6 μm, [x = 5.0 ± 0.3 × 4.3 ± 0.2, Q = 1.0–1.3, Q_m_ = 1.2 ± 0.1, n = 20], globose, broadly ellipsoid, smooth, thick-walled, brown. *Basidia* 24.6–38.6 × 8.8–11.6 μm, clavate, hyaline, 4-spored, smooth. *Cheilocystidia* 15–37.4 × 5.4–19.7 μm, smooth, hyaline, clavate mostly, broadly clavate or capitate with long narrow stipe, and base septate. *Pleurocystidia* absent. *Pileipellis* a cutis composed of hyphae of 2.7–6.8 μm in diam., smooth, cylindrical, brown, slightly constricted at septa.

*Habitat*: solitary or gregarious on plateau grassland.

*Other specimens examined*: Bangda Grassland, Basu County, Tibet, China, 4 August 2012, collected by Zhao Rui-Lin, *ZRL2012652* (HMAS275782), *ZRL2012653* (HMAS275795); Wulashan Grassland, Mangkang County, Tibet, China, 5 August 2012, collected by Dong Xin-Yu, *ZRL2012658* (HMAS275749).

Note: *Agaricus globosporus* is represented by a clade composed of two specimens (*ZRL2012652*, *ZRL2012656*) under the fully support of 1.0/100 BS/PP values (Figs 1–2). This new species clustered with *A*. *armandomyces*, *A*. *kerriganii* and *A*. *edmondoi* under the support of 0.9/- PP/BS values in clade I (Figs 1–2). In morphology, *A*. *globosporus* resembles *A*. *brunneolus* (J.E. Lange) Pilát and *A*. *comtulus* Fr., because they all have brown pileus, cylindrical to clavate stipe, and similar cheilocystidia in shape and size. However *A*. *globosporus* has near globose basidiospores (Q = 1.0–1.3), while those of *A*. *brunneolus* and *A*. *comtulus* are broadly ellipsoid (Q = 1.2–1.6)^4^.

**Figure 13:**
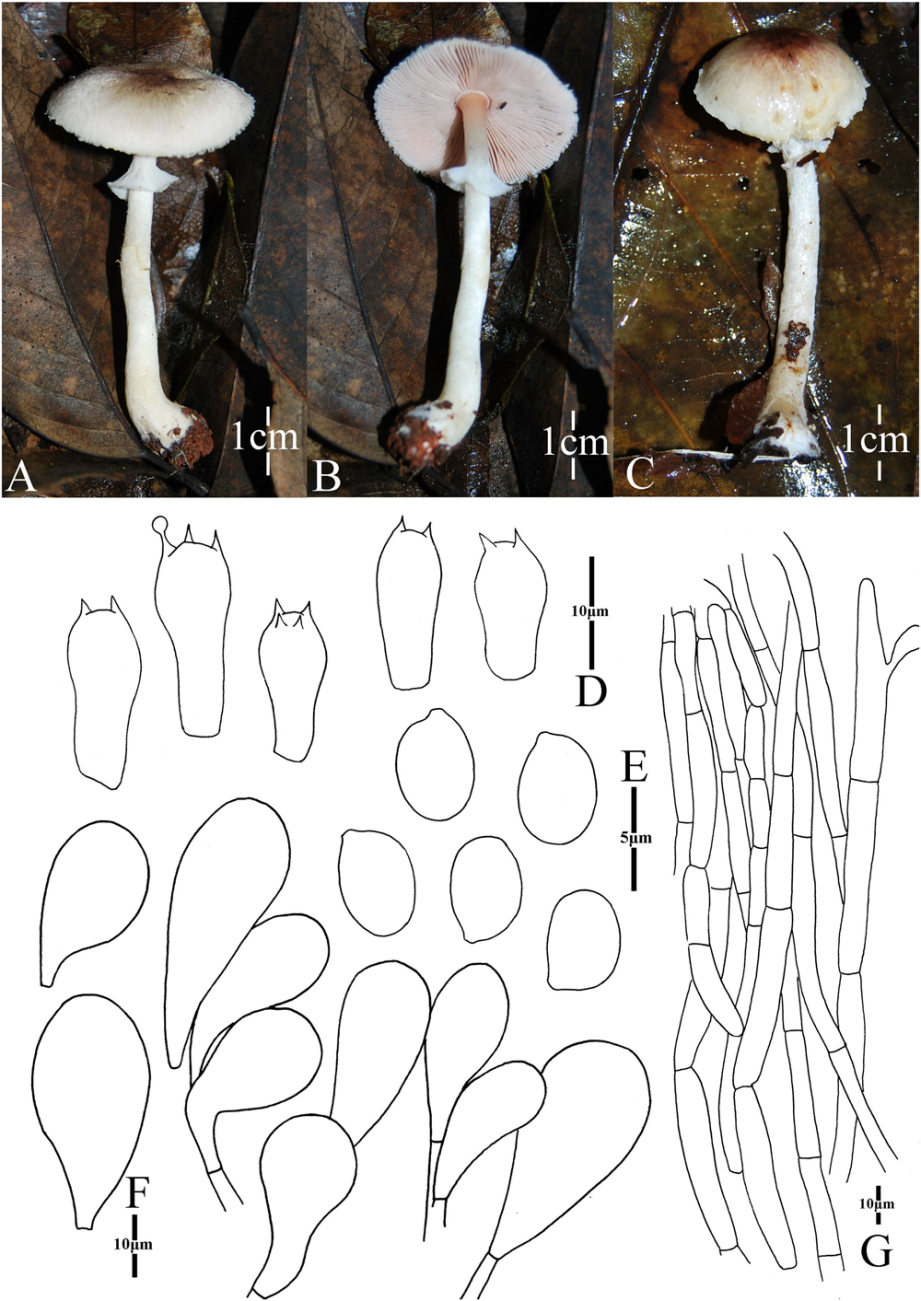
Morphology of *Agaricus mangaoensis* (*ZRL2010056*, holotype), (**A**–**C**): Basidiome in field (**A**,**B**): *ZRL2010056*; (**C**): *ZRL2010073*), (**D**): Basidia, (**E**): Basidiospores, (**F**): Cheilocystidia, and (**G**): Pileipellis hyphae.

10. *Agaricus mangaoensis* M.Q. He & R.L. Zhao. *sp*. *nov*.; Fig. 13

Fungal Names: FN570350

Faceoffungi Number: FoF 02930

*Etymology*: the epithet “*mangao*” refers to the region Mangao County from where the holotype was collected.

*Holotype*: Manda Village, Mangao, Xishangbanna, Yunnan Prov., China, 24 July 2010, collected by Zhao Rui-Lin, *ZRL2010056* (HMAS275777, holotype).

Original description: *Pileus* 22–30 mm in diam., convex, applanate with subumbo; margin straight, exceeding; surface dry, covered by fibrils, reddish brown, purplish brown at disc, and fading into white towards margin, appressed, turn reddish purple in wet. *Context* 1–2 mm thick, flesh, white. *Lamellae* 2–3 mm broad, free, crowded, pink or pinkish brown first, then brown in age, edge white, crenate, intercalated with lamellulae. *Annulus* 2–3 mm in diam., single, fragile, membranous, white, pendant, smooth on both sides. *Stipe* 50 × 2–3 (5–7 at base) mm, white, hollow, cylindrical, bulbous, surface above the annulus smooth, below fibrillose, white, always with rhizomorphs. Odour of almonds. Basidiome flavescent when touching, bruising and cutting.

KOH reaction: positive yellow. Schäffer’s reaction: positive, reddish orange on dry specimen.

*Basidiospores* 5.0–6.2 × 3.3–3.9 μm, [x = 5.5 ± 0.3 × 3.6 ± 0.2, Q = 1.5–1.7, Q_m_ = 1.5 ± 0.1, n = 20], elongate, smooth, thick-walled, brown. *Basidia* 11–16.0 × 6.0–8 μm, clavate, hyaline, 4-spored, smooth. *Cheilocystidia* 15.8–43.0 × 9.6–23 μm, smooth, single, clavate, broadly clavate, hyaline or containing yellow pigments. *Pleurocystidia* absent. *Pileipellis* a cutis composed of hyphae of 3.7–15 μm in diam., smooth, cylindrical, brown, slightly constricted at septa in some cases.

*Habitat*: solitary on soil in forest.

*Other specimens examined*: Nangongshan Village, Xishangbanna, Yunnan Prov., China, 25 June 2010, collected by Zhao Rui-Lin, *ZRL2010073* (HMAS275742), *ZRL2010078* (HMAS275743).

Notes: This new species is represented by specimen *ZRL2010056*. In phylogeny, this species is sister to the unnamed specimen *ZRL20151437* under the fully support of 1.0/100 PP/BS values in the clade IX (Figs 1–2). In the morphology, *A*. *mangaoensis* is easily separated from specimens *ZRL20151437* because the latter has middle-sized basidiome. Some species of section *Minores* have tiny or small basidiome and reddish brown, purple brown caps, such as the known species *A*. *gemloides* ^5^, *A*. *purpurellus* ^4^ and *A*. *parvibicolor* ^19^, but their phylogenetic positions are far from *A*. *mangaoensis*, and nested in clade I and clade II respectively (Figs 1–2). In this study we introduce another new species *A*. *minorpurpureus*, which is morphologically similar to *A*. *mangaoensis* in the field. However, *A*. *mangaoensis* has elongate basidiospores and those of *A*. *minorpurpureus* are broadly ellipsoid (Q = 1.4–1.6). Based on the phylogenetic and morphological analysis, we proposed this species as new to the science. This new species is characterized by its small basidiome (less than 30 mm in diam. of pileus), reddish brown fibrils on the cap, stipe cylindrical with bulbous base and clavate cheilocystidia.

**Figure 14:**
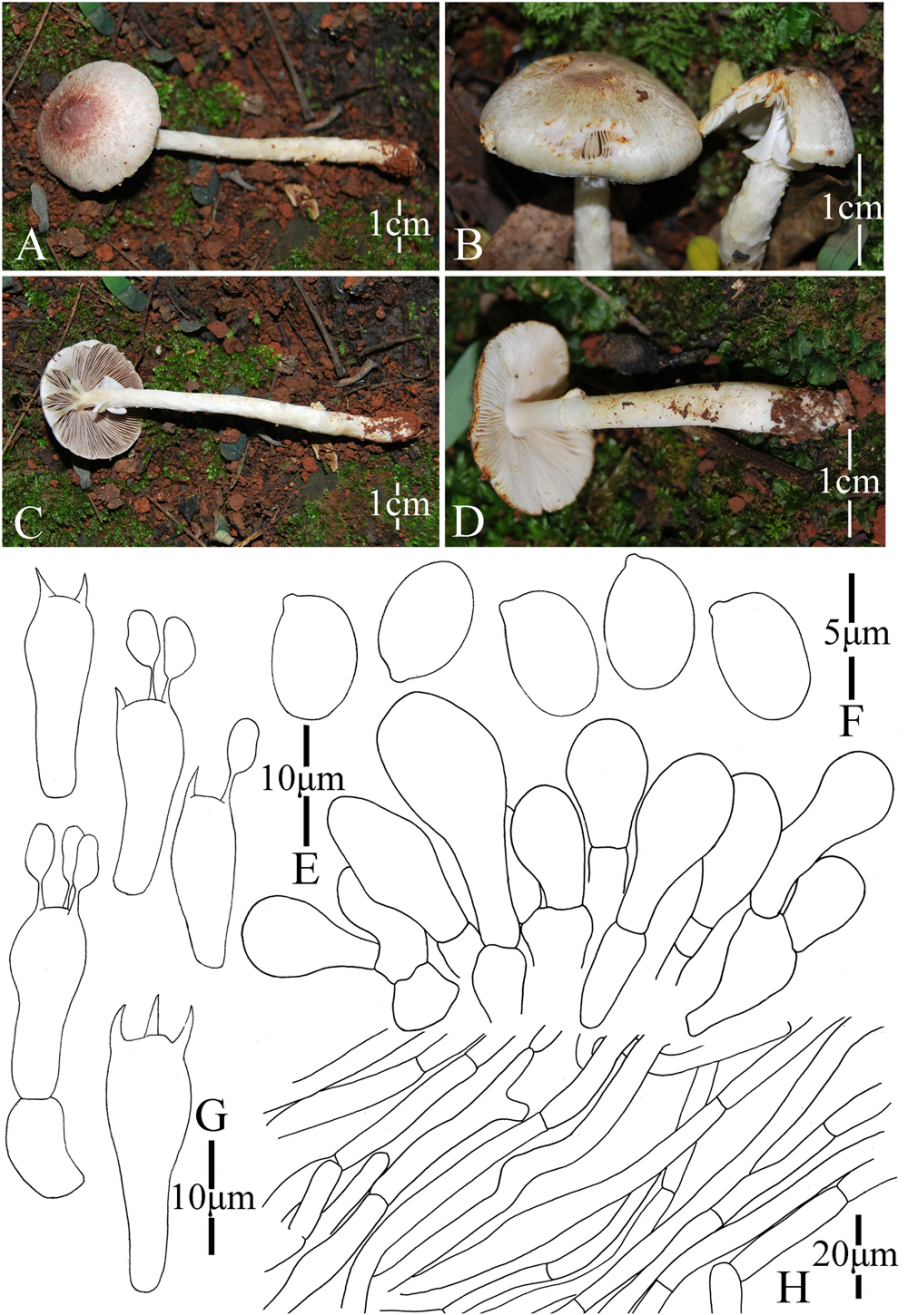
Morphology of *Agaricus microviolaceus* (*ZRL2012718*, holotype), (**A**–**D**): Basidiome in field (**A**,**C**: *ZRL2012718*; (**B**): *ZRL2012714*; (**D**): *ZRL2012717*), (**E**): Cheilocystidia, (**F**): Basidiospores, (**G**): Basidia, and (**H**): Pileipellis hyphae.

11. *Agaricus microviolaceus* M.Q. He & R.L. Zhao *sp*. *nov*.; Fig. 14

Fungal Names: FN570343

Faceoffungi Number: FoF 02931

*Etymology*: the epithet “*micro*” means small, “*violaceus*” means purple, refers to the small basidiome and the purple pileus.

*Holotype*: Dalongkou forest park, Yimen County, Yunnan Prov., China, 17 August 2012, collected by Zhou Jun-Liang, *ZRL2012718* (HMAS275791, holotype).

Original description: *Pileus* 18–26 mm in diam., convex, plane; disc slightly umbonate; margin straight, with appendiculate remains of universal veil; surface dry, covered by fibrils and forming fibrillose scales, denser at disc, scattered towards the margin, appressed, purple, reddish brown, fading into white towards margin. *Context* 1–2 mm thick, flesh, white. *Lamellae* 2–3 mm broad, free, crowded, pink or pinkish brown firstly, then brown, edge crenate, intercalated with lamellulae. *Annulus* 3 mm in diam., single, membranous, white, pendant, upper surface smooth, lower surface fibrillose. *Stipe* 30–66 × 2–3 (3–4 at base) mm, white, hollow, cylindrical, surface dry, surface below the annulus fibrillose. Odour of almonds. Basidiome strongly yellow then orange brown after several minutes when touching, bruising and cutting.

KOH reaction: positive yellow. Schäffer’s reaction: positive, reddish orange on dry specimen.

*Basidiospores* 4.6–6.2 × 3.0–3.6 μm, [x = 5.2 ± 0.4 × 3.3 ± 0.2, Q = 1.3–1.8, Q_m_ = 1.6 ± 0.1, n = 20], ellipsoid, elongate, smooth, thick-walled, brown. *Basidia* 11.8–18.2 × 5.4–7.0 μm, clavate, hyaline, 4-spored, smooth. *Cheilocystidia* 7.6–26.4 (−47.8) × 5.6–10.6 μm, smooth, single, clavate mostly, broadly clavate, hyaline or containing yellow pigments. *Pleurocystidia* absent. *Pileipellis* a cutis composed of hyphae of 4.5–10 μm in diam., smooth, cylindrical, hyaline, having yellow or light brown pigments.

*Habitat*: solitary on soil in forest.

*Other specimens examined*: Dalongkou, Yimen County, Yunnan Prov., China, 17 August 2012, collected by Xie Meng, *ZRL2012716* (HMAS275790), *ZRL2012717* (HMAS275792), *ZRL2012714* (HMAS273969).

Notes: In our phylogeny analysis, *A*. *microviolaceus* is represented by three specimens which nest together with the support of 1.0/100 PP/BS values. *Agaricus microviolaceus* cluster with other three unnamed specimens (*ZRL3056*, *ZRL2011039*, and *LD201252*) and composed of clade VI with the support of 1.0/100 PP/BS values (Figs 1–2). This new species is characterized by its slender basidiome, purple fibrillose scales on the cap and clavate cheilocystidia which contains yellow pigments. This combination of morphological characters make it similar to another introduced new species *A*. *mangaoensis* in this study and *A*. *gemloides* ^5^. In the field, this new species can separated from *A*. *mangaoensis* by the latter has much darker fibrils on the cap and bulbose stipe. Compared with *A*. *gemloides*, the capitated cheilocystidia of *A*. *gemloides* is distinct morphological character to separate from *A*. *microviolaceus*.

**Figure 15:**
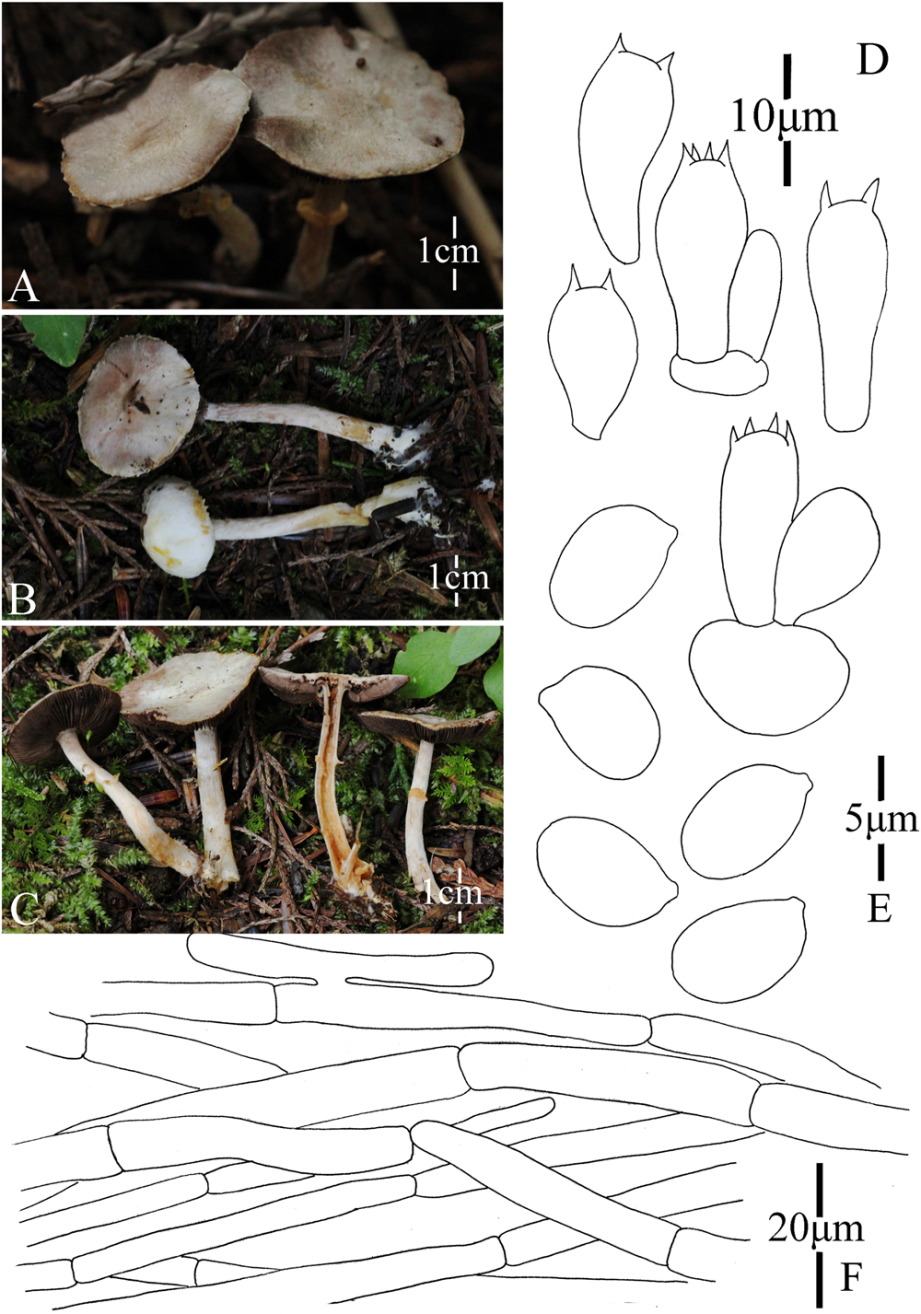
Morphology of *Agaricus pseudopallens* (*ZRL20151552*, holotype), (**A**–**C**): Basidiome in field (**A**,**B**): *ZRL20151549*; (**C**): *ZRL20151552*), (**D**): Basidia (**E**): Basidiospores, and (**F**): Pileipellis hyphae.

12. *Agaricus pseudopallens* M.Q. He & R.L. Zhao *sp*. *nov*.; Fig. 15

Fungal Names: FN570344

Faceoffungi Number: FoF 02932

*Etymology*: the epithet of “*pseudopallens*” refers to the morphology of this species is similar with *A*. *pallens*.

*Holotype*: Caoyutang Forest Park, Jingning County, Zhejiang Prov., China, 19 August 2015, collected by Su Sheng-Yu, *ZRL20151552* (HMAS275786, holotype).

Original description: *Pileus* 23–38 mm in diam., convex, plane; disc flat; margin straight; surface dry, covered by fibrils, white or slightly gray, forming fibrillose scales at disc, appressed or recovered. *Context* 2 mm thick, flesh, white or gray. *Lamellae* up to 2 mm broad, free, crowded, pink or grayish brown firstly, then brown in age. *Annulus* single, fragile, membranous, white, pendant, smooth, turn to yellow when bruised or dry. *Stipe* 23–50 × 3–3.5 (7–9 at base) mm, hollow, cylindrical, with short rhizomorphs, surface dry, white or gray, smooth above the annulus, below fibrillose. Odour of aniseed. Basidiome strongly yellow when touching, especially on the stipe and cap. Discoloration yellow firstly, then yellowish brown after few minutes on cutting.

KOH reaction: positive yellow. Schäffer’s reaction: positive, reddish orange on dry specimen.

*Basidiospores* 5.0–5.8 × 3.0–3.6 μm, [x = 5.4 ± 0.2 × 3.3 ± 0.1, Q = 1.5–1.8, Q_m_ = 1.6 ± 0.1, n = 20], ellipsoid, elongate, smooth, thick-walled, brown. *Basidia* 13.2–19.0 × 5.4–7.2 μm, long clavate, hyaline, 4-spored, smooth. *Cheilocystidia* absent. *Pleurocystidia* absent. *Pileipellis* a cutis composed of hyphae of 3.2–9 μm in diam., smooth, cylindrical, hyaline or yellow brown, slightly constricted at septa.

*Habitat*: solitary on soil in forest.

*Other specimens examined*: Caoyutang Forest Park, Jingning County, Zhejiang Prov., China, 19 August 2015, collected by He Mao-Qiang *ZRL20151549* (HMAS275788); Cangshan Mountain, Dali, Yunnan Prov., China, 29 July 2014, collected by He Mao-Qiang *ZRL2014154B* (HMAS275826).

Notes: *Agaricus pseudopallens* is represented by a clade which is composed of two specimens with the support of 1.0/78 PP/BS values. This species is sister to *A*. *pallens*, then nest with *A*. *heinemannianus* Esteve-Rav. in clade I (Figs 1–2). In morphology, *A*. *heinemannianus* is easily distinguished from *A*. *pseudopallens* in the field by its distinct reddish purple fibrillose scales on the pileus. Under the microscope, the basidiospores of *A*. *pseudopallens* (Q_m_ = 1.6) are narrower than those of *A*. *heinemannianus* (Q_m_ = 1.4) (Parra 2013). *Agaricus pseudopallens* is phylogenetic and morphologically similar to *A*. *pallens* mostly, because both are white cap. However *A*. *pallens* has abundant of cheilocystidia^4^, while *A*. *pseudopallens* is lacking cheilocystidia. Based on the distinct morphological and phylogenetic features, we proposed *A*. *pseudopallens* as a new species, and this species is characterized by its nearly white and smooth pileus, elongate ellipsoid basidiospores and absence of cheilocystidia.

**Figure 16:**
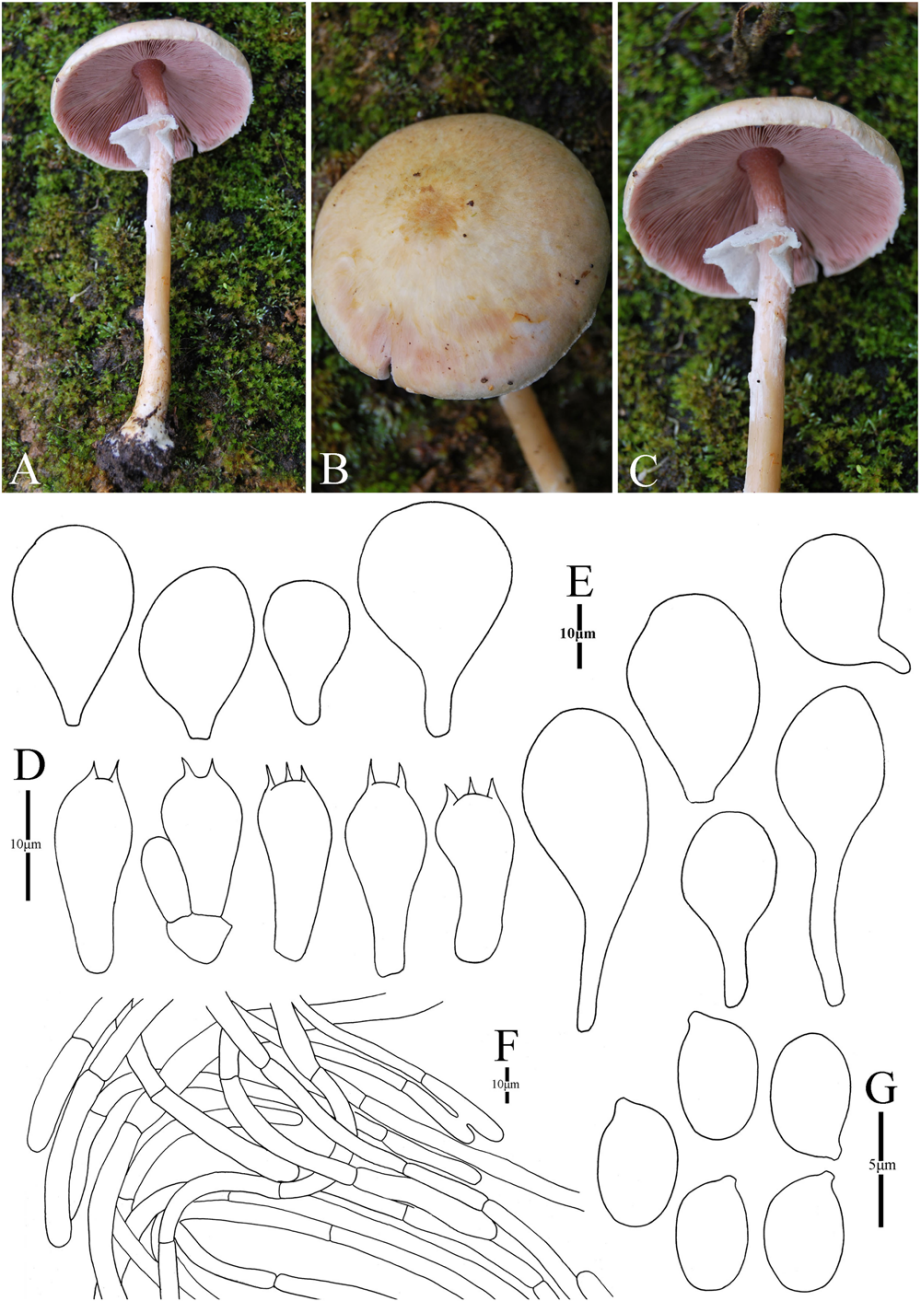
Morphology of *Agaricus elongatestipes* (*ZRL2013271*, holotype), (**A**–**C**): Basidiome in field (*ZRL2013271*), (**D**): Basidia, (**E**): Cheilocystidia, (**F**): Pileipellis hyphae, and (**G**): Basidiospores.

13. *Agaricus elongatestipes* M.Q. He & R.L. Zhao *sp*. *nov*.; Fig. 16

Fungal Names: FN570345

Faceoffungi Number: FoF 02933

*Etymology*: the epithet “*elongate*” refers to the slender basidiome of this species.

*Holotype*: Laifengshan mountain, Tengchong County, Yunnan Prov., China, 8 July 2013, collected by Su Sheng-Yu, *ZRL2013271* (HMAS275773, holotype).

Original description: *Pileus* 55–58 mm in diam., convex; margin straight, slightly exceeding; surface covered by fibrils, ocherous-yellow, appressed, denser at disc, scattered towards margin, easily rubbed by raindrop; background white, turn to red in wet. *Context* 1–2 mm thick, flesh, white. *Lamellae* 4–4.5 mm broad, free, crowded, pink, edge even, entire, intercalated with lamellulae. *Annulus* 12–22 mm in diam., single, fragile, membranous, white, pendant, smooth on both sides. *Stipe* 105–110 × 5–8 (14–15 at base) mm, cylindrical, bulbous at base, with rhizomorphs, hollow, surface above the annulus smooth, below fibrillose, white. Odour of almonds. Basidiome flavescent when touching, bruising and cutting.

KOH reaction: positive yellow. Schäffer’s reaction: positive, reddish orange on dry specimen.

*Basidiospores* 4.6–5.4 × 3.0–3.5 μm, [x = 5.0 ± 0.2 × 3.3 ± 0.2, Q = 1.4–1.7, Q_m_ = 1.5 ± 0.1, n = 20], ellipsoid, elongate, smooth, thick-walled, brown. *Basidia* 12.8–20.5 × 5.9–7 μm, clavate, hyaline, 4-spored, smooth. *Cheilocystidia* 16–33 × 10– 18.5 μm, smooth, clavate, pyriform or capitate with long narrow stipe, sometimes globose, containing yellow pigments. *Pleurocystidia* absent. *Pileipellis* a cutis composed of hyphae of 2.8–11.2 μm in diam., smooth, cylindrical, hyaline or light brown, constricted at septa.

*Habitat*: solitary on soil in forest.

*Other specimens examined*: Laifengshan, Tengchong County, Yunnan Prov., China, 8 July 2013, 8 July 2013, collected by Yu Qing-Hua, *ZRL2013265* (HMAS275772).

Notes: In phylogeny analysis *A*. *elongatestipes* is represented by two specimens and clade together under the statistic support of 1.0/92 PP/BS in clade XI (Figs 1–2). This new species is characterized by its slender and related long stipe, weak fibrils on the cap and capitate cheilocystidia which contains yellow pigments. In the phylogeny, *A*. *elongatestipes* is sister to the unnamed specimen *ZRLLD013* (Figs 1–2), but they are obviously different species because the cheilocystidia of *ZRLLD013* are globose or broadly clavate.

**Figure 17:**
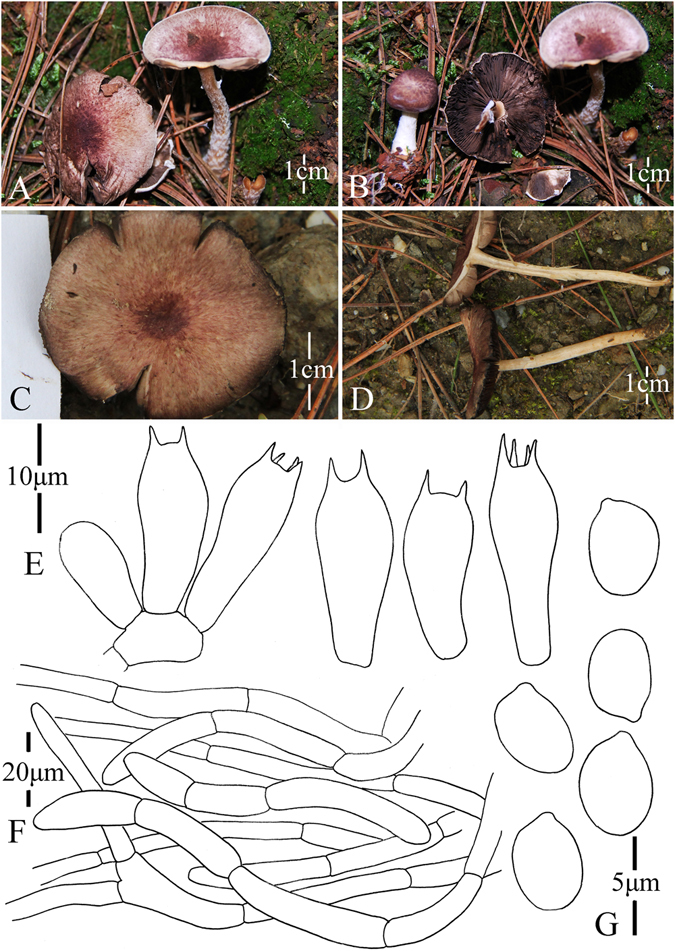
Morphology of *Agaricus pseudopurpurellus* (*ZRL2014063*, holotype), (**A**–**D**): Basidiome in the field (**A**,**B**: *ZRL2012012*; (**C**,**D**): *ZRL2014063*), (**E**): Basidia (**F**): Pileipellis hyphae, and (**G**): Basidiospores.

14. *Agaricus pseudopurpurellus* M.Q. He & R.L. Zhao *sp. nov.*; Fig. 17

Fungal Names: FN570346

Faceoffungi Number: FoF 02934

*Etymology*: refers to this new species is morphologically similar with *A*. *purpurellus*.

*Holotype*: Dali city, Yunnan Prov., China, 25 June 2014, collected by Weili, *ZRL2014063* (HMAS275745, holotype).

Original description: *Pileus* 20–30 mm in diam., parabolic when young, then convex, plane finally, disc flat; margin straight, slightly uplifted when mature; surface dry, covered by fibrils at the whole cap; forming fibrillose scales at disc, purple, reddish brown, and scattered towards the margin, appressed. *Context* up to 1 mm thick, flesh, white. *Lamellae* 1.5–3 mm broad, free, crowded, ventricose, brown, edge even. *Annulus* 2 mm in diam., single, fragile, membranous, white, pendant, smooth at both sides. *Stipe* 42–45 × 3–4 (3–8 at base) mm, white, hollow, long clavate, surface dry, smooth above the annulus, with fibrillose scales below the annulus. Odour of almonds. Basidiome flavescent when touching, bruising and cutting.

KOH reaction: positive yellow. Schäffer’s reaction: positive, reddish orange on dry specimen.

*Basidiospores* 4.4–5.4 × 3.0–3.6 μm, [x = 4.8 ± 0.2 × 3.3 ± 0.2, Q = 1.3–1.6, Q_m_ = 1.5 ± 0.1, n = 20], ellipsoid, broadly ellipsoid, smooth, thick-walled, brown. *Basidia* 11.5–16.0 × 5.1–7.6 μm, clavate, hyaline, 4-spored, smooth. *Cheilocystidia* absent. *Pleurocystidia* absent. *Pileipellis* a cutis composed of hyphae of 2.3–6 μm in diam., smooth, cylindrical, light brown, slightly constricted at septa.

*Habitat*: solitary on soil in forest.

*Other specimens examined*: Yeyahu Park, Kunming, Yunnan Prov., China, 30 June 2012, collected by Zhao Rui-Lin, *ZRL2012012* (HMAS273936).

Notes: In phylogeny *A*. *pseudopurpurellus* is represented by the only specimen *ZRL2014063*. In the MCC tree (Fig. 2) this species is sister to all species of section *Minores* with a distinct phylogenetic position; while in the ML tree (Fig. 1), it is sister to clades XI and XII but without statistic supports. In morphology, the *A*. *purpurellus* is the most morphologically similar species to the proposed new species, because both have the small-sized basidiome and covered by purple scales on the cap^4^. But *A*. *purpurellus* has clavate or capitate cheilocystidia, while the cheilocystidia of *A*. *pseudopurpurellus* is absent. Furthermore, the phylogenetic analysis shows they are different species. Then we propose *A*. *pseudopurpurellus* as a new species.

**Figure 18:**
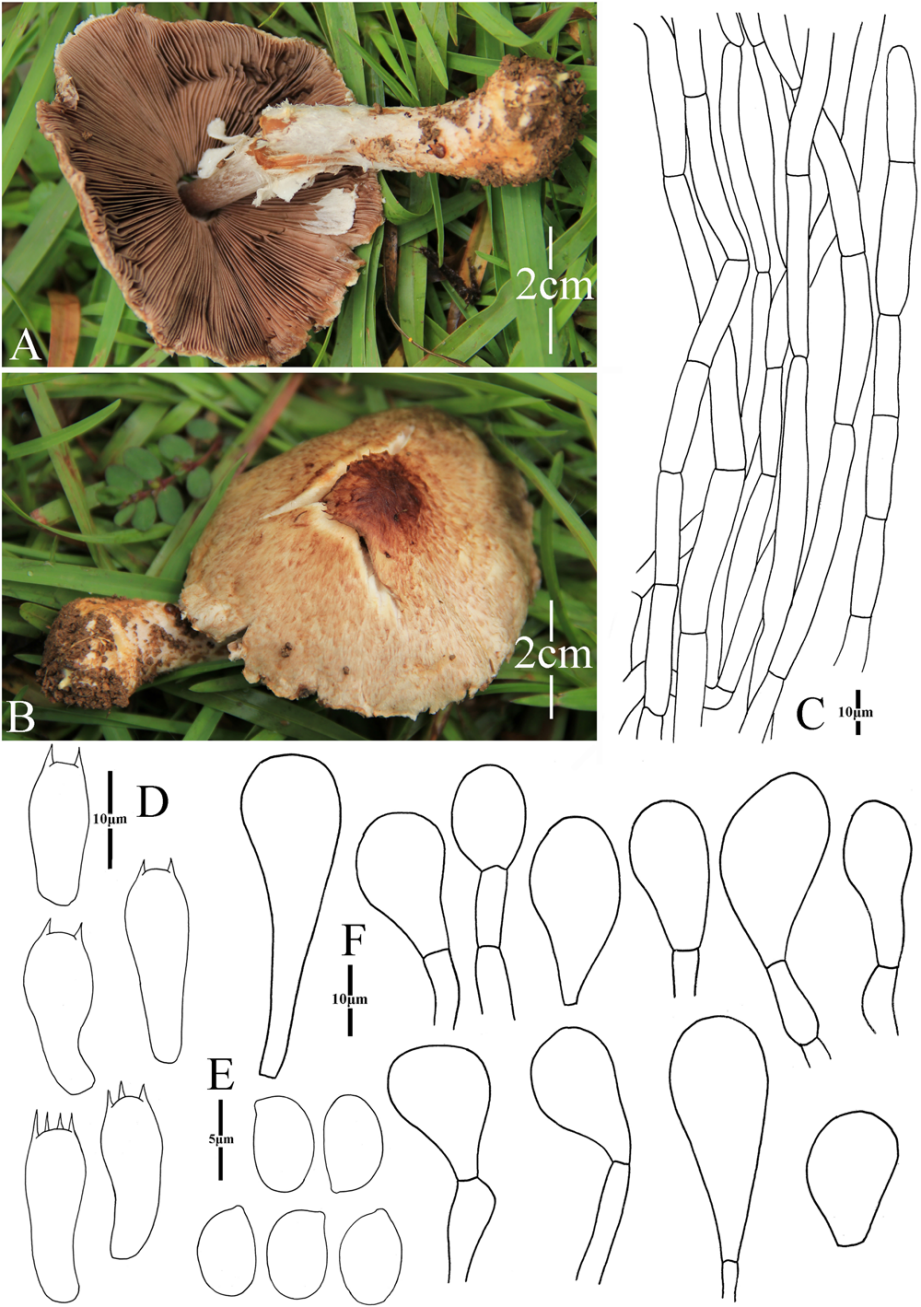
Morphology of *Agaricus rufuspileus* (*ZRL2014140*, holotype), (**A**,**B**): Basidiome in field (*ZRL2014140*), (**C**): Pileipellis hyphae, (**D**): Basidia, (**E**): Basidiospores, and (**F**): Cheilocystidia.

15. *Agaricus rufuspileus* M.Q. He & R.L. Zhao *sp*. *nov*.; Fig. 18

Fungal Names: FN570347

Faceoffungi Number: FoF 02935

*Etymology*: the epithet “*rufus*” means reddish brown, refers to the reddish-brown pileus.

*Holotype*: Yangbi County, Dali, Yunnan Prov., China, 28 July 2014, collected by He Mao-Qiang, *ZRL2014140* (HMAS275780, holotype).

Original description: *Pileus* 49–60 mm in diam., convex, plane, disc subumbonate, margin straight, exceeding; surface dry, covered by fibrillose scales, brown or reddish brown, appressed or recurved, triangular, denser at disc and scattered towards the margin. *Context* 2–4 mm thick at disc, flesh, white. *Lamellae* 4–5 mm broad, free, crowded, brown. *Annulus* 5–16 mm in diam., single, membranous, white, pendant, fragile, smooth on both sides. *Stipe* 55–67 × 4–6 (9–18 at base) mm, white, hollow, cylindrical, distinct bulbous at base, surface smooth above the annulus, with white fibrils below the annulus. Odour of almonds. Basidiome flavescent when touching and cutting.

KOH reaction: positive yellow. Schäffer’s reaction: positive, reddish orange on dry specimen.

*Basidiospores* 5.3–6.3 (−6.7) × 3.2–4.0 μm, [x = 5.8 ± 0.3 × 3.7 ± 0.2, Q = 1.4–1.7, Q_m_ = 1.6 ± 0.1, n = 20], ellipsoid or elongate, smooth, thick-walled, brown. *Basidia* 12.4–19.1 × 5.9–8.1 μm, clavate, hyaline, 4-spored, smooth. *Cheilocystidia* 21.7–39 × 10.5–16 μm, single, smooth, hyaline, broadly clavate mostly, or broadly clavate, globose, some septate at base, containing yellow pigments or not, *Pleurocystidia* absent. *Pileipellis* a cutis composed of hyphae of 4.5–7 μm in diam., smooth, cylindrical, light brown, yellow, constricted at septa sometimes.

*Habitat*: scattered on soil in forest.

*Other specimens examined*: Yangbi County, Dali, Yunnan Prov., China, 28 July 2014, collected by Bai Xun-Ming, *ZRL2014146* (HMAS275779); same location, 28 July 2014, collected by Su Sheng-Yu, *ZRL2014147* (HMAS275765), *ZRL2015145* (HMAS275781); Pingshan Village, Longchuan County, Yunnan Prov., China, 23 July 2013, collected by Zhou Jun-Liang, *ZRL2013480* (HMAS275753).

Note: In phylogenetic tree there are six specimens represented *A*. *rufuspileus*, and all clustered together under the support of 1/61 PP/BS values in clade IV (Figs 1–2). The known species *A*. *brunneolus* has reddish brown scales on the cap too, but it differs in its larger sized cap (30–110 mm in diam.) than those of *A*. *rufuspileus* ^4^. *Agaricus marisae* L.A. Parra & Callac is the most similar species with *A*. *rufuspileus* in morphology, however, the cheilocystidia of *A*. *marisae* is clavate and elongate with the width of 5–10 μm, while those of *A*. *rufuspileus* is broadly clavate with the width of 10.5–16.0 μm^4^. In phylogeny, those two known species are nested in clade I where is far from the proposed new species *A*. *rufuspileus* (Figs 1–2). *Agaricus rufuspileus* is characterized by its reddish brown cap and clavate, broadly clavate cheilocystidia.

**Figure 19:**
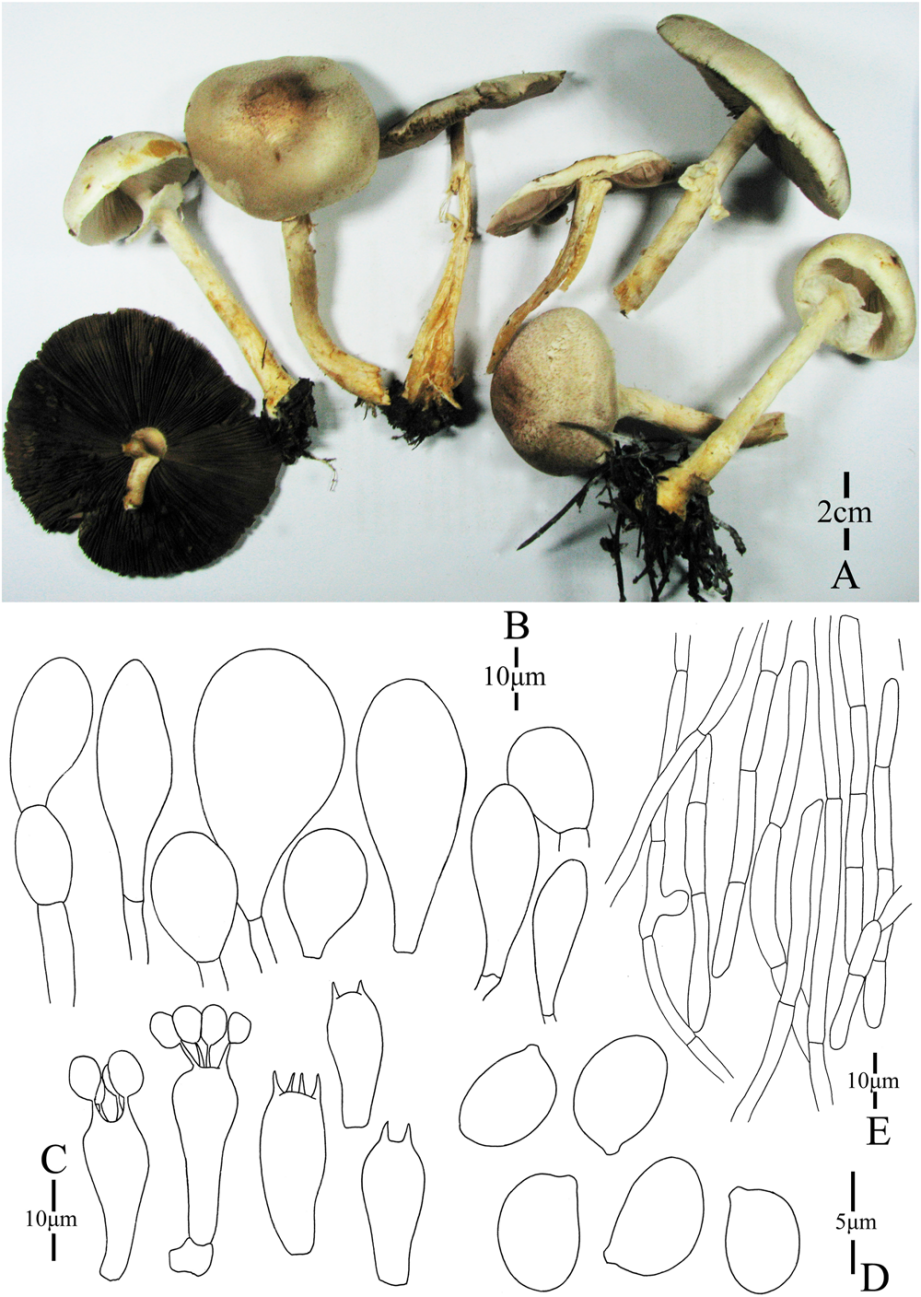
Morphology of *Agaricus neimengguensis* (*ZRL20151845*, holotype), (**A**): Basidiome in field (*ZRL20151845*), (**B**): Cheilocysitidia, (**C**): Basidia, (**D**): Basidiospores, and (**E**): Pileipellis hyphae.

16. *Agaricus neimengguensis* M.Q. He & R.L. Zhao *sp*. *nov*.; Fig. 19

*Fungal Names*: FN570348

*Faceoffungi Number*: FoF 02936

*Etymology*: the epithet “*neimenggu*” refers to the province Neimenggu where the holotype was collected.

*Holotype*: Sanjiao Mountain, Aershanyiershi Town, Neimenggu Prov., China, 23 Aug 2015, collected by Dai Rong-Chun, *ZRL20151845* (HMAS254648, holotype).

Original description: *Pileus* 25–71 mm in diam., parabolic when young, then convex, finally plane, with umbo at disc; margin entire, slightly exceeding and appendiculate when young; surface dry, covered by fibrils at the whole cap; forming tiny fibrillose scales at disc, brown or reddish brown, triangular, appressed, then fading towards the margin. *Context* 2–6 mm thick, flesh, white, gray. *Lamellae* 2–4 mm broad, free, crowded, pink or pinkish brown firstly, then brown, edge even, narrow to broad, intercalated with lamellulae. *Annulus* 2–4 mm in diam., single, membranous, smooth on both sides, pendant, persistent, white, turning yellow when dry or old. *Stipe* 42–82 × 5–7 (5–8 at base) mm, white, hollow, long cylindrical, surface dry, above the annulus smooth, below slightly fibrillose. Odour of bitter almonds or aniseed. Basidiome flavescent immediately, then orange brown after few minutes when touching, bruising and cutting.

KOH reaction: positive yellow. Schäffer’s reaction: positive, reddish orange on dry specimen.

*Basidiospores* 5.0–6.1 × 3.4–4.2 μm, [x = 5.5 ± 0.3 × 3.8 ± 0.2, Q = 1.4–1.5, Q_m_ = 1.5 ± 0.0, n = 20], ellipsoid, smooth, thick-walled, brown. *Basidia* 15.2–21.9 × 6.4–8.4 μm, clavate, hyaline, 4-spored, smooth. *Cheilocystidia* 17.1–42.0 × 8.2–24.7 μm, smooth, clavate mostly, broadly clavate, hyaline, rarely clavate, occasionally 1-sepat at the base, always containing yellow pigments. *Pleurocystidia* absent. *Pileipellis* a cutis composed of hyphae of 4.6–7.6 μm in diam., smooth, cylindrical, brown, slightly constricted at some septa.

*Habitat*: solitary on soil in forest.

*Other specimens examined*: Sanjiao Mountain, Aershanyiershi Town, Neimenggu Prov., China, 23 Aug 2015, collected by Dai Rong-Chun, *ZRL20151831* (HMAS275799); same location, 23 Aug 2015, collected by Li Guo-Jie, *ZRL20151841* (HMAS280111); Honghuaerji Natural Reserve, Hu Lunbeier, Neimenggu Prov., China, 22 Aug 2015, collected by Li Guo-Jie, *ZRL20151815* (HMAS275800).

Notes: Five specimens represented as *A*. *neimengguensis* and cluster together with the support of 1.0/100 PP/BS values (Figs 1–2). *Agaricus neimengguensis* and the unnamed specimen Vellinga2360 composed of clade VII, which located at an isolated phylogenetic position (Figs 1–2). This new species is characterized by weak fibrils at the pileus and cheilocystidia always containing yellow pigments.

The following three species are introduced as new records for China.

**Figure 20:**
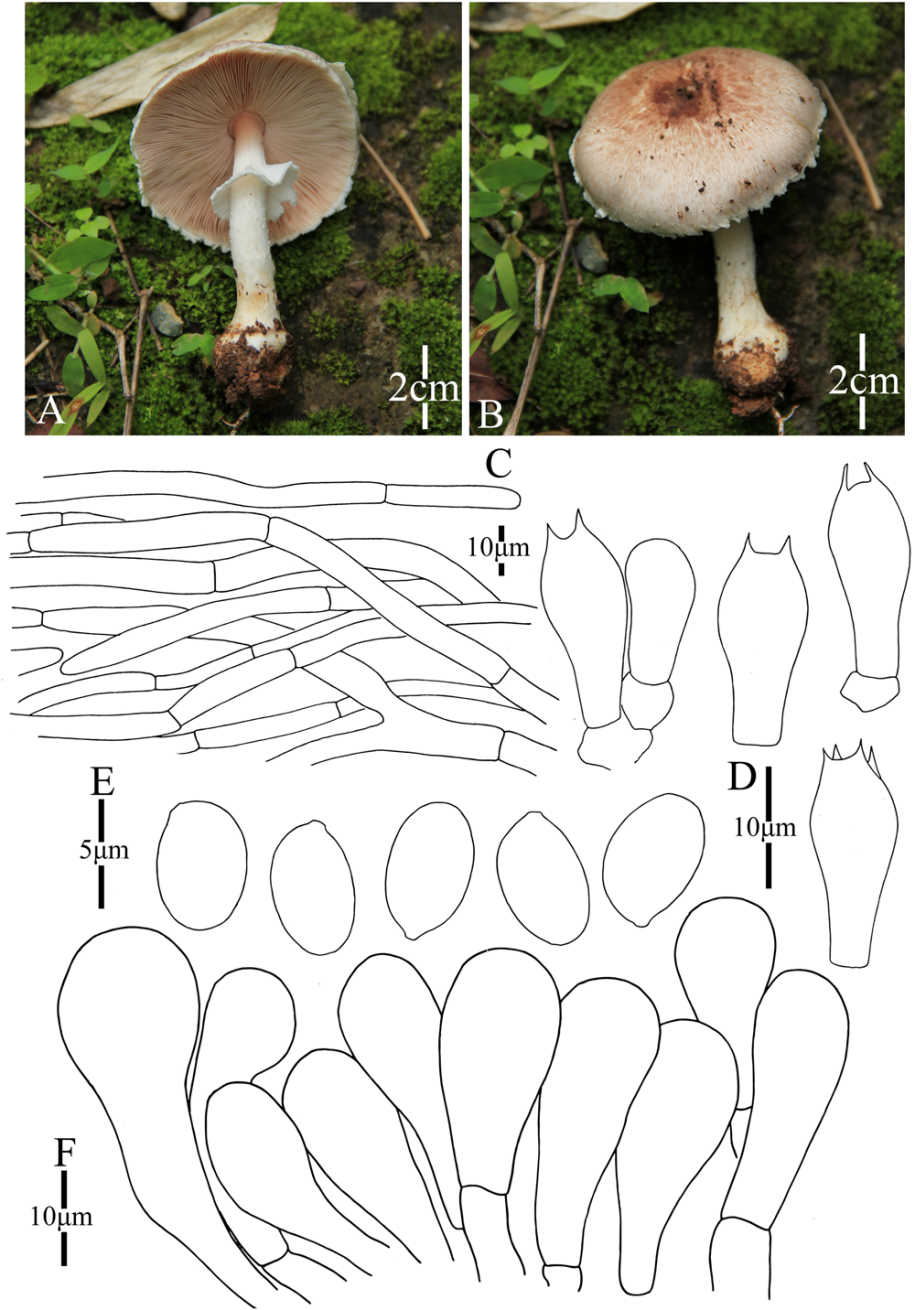
Morphology of *Agaricus patris* (**A**,**B**): Basidiome in field (*ZRL2014134*), (**C**): Pileipellis hyphae, (**D**): Basidia, (**E**): Basidiospores, and (**F**): Cheilocystidia.

1. *Agaricus patris* L.J. Chen, Callac, K.D. Hyde & R.L. Zhao, *Persoonia*, **38**, 2017: 170–196. Figure 20

*Pileus* 54 mm in diam., convex with slightly truncated disc, applanate; margin entire, slightly exceeding and with the appendiculate remains of universal veil; surface dry, covered by fibrils completely; forming fibrillose scales, reddish brown, triangular, appressed, denser at disc, scattered radially towards the margin. *Context* 5 mm thick, flesh, white. *Lamellae* 5 mm broad, free, crowded, pink or pinkish brown, edge even, normal, intercalated with lamellulae. *Annulus* 10 mm in diam., single, membranous, smooth on both sides, pendant, persistent, white. *Stipe* 62 × 5 (12 at base) mm, white, hollow, cylindrical with bulbous base, surface dry, above the annulus smooth, below slightly fibrillose. Odour of bitter almonds. Basidiome flavescent immediately on touching, bruising and cutting.

KOH reaction: positive yellow. Schäffer’s reaction: positive, reddish orange on dry specimen.

*Basidiospores* 5.4–6.4 × 3.6–4.2 μm, [x = 5.9 ± 0.3 × 3.8 ± 0.2, Q = 1.4–1.8, Q_m_ = 1.5 ± 0.1, n = 20], ellipsoid, smooth, thick-walled, brown. *Basidia* 14.9–18.9 × 5.9–7.8 μm, clavate, hyaline, 4-spored, smooth. *Cheilocystidia* 12–44.6 × 7.3–14 μm, smooth, clavate, broadly clavate, hyaline, some containing yellow pigments. *Pleurocystidia* absent. *Pileipellis* a cutis composed of hyphae of 5–8 μm in diam., smooth, cylindrical, brown, yellowish brown, slightly constricted at septa.

*Habitat*: solitary on the side of road.

*Specimens examined*: Sangbulao Village, Dali, Yunnan Prov., China, collected by He Mao-Qiang, *ZRL2014134* (HMAS275746).

Notes: *Agaricus patris* recently described from Thailand^10^. The morphological characters of this specimen is identical with the original description^10^. In phylogenetic analysis (Figs 1–2), our specimen also nest with the type specimen of *A*. *patris* well. Here this species is firstly reported for China.

**Figure 21:**
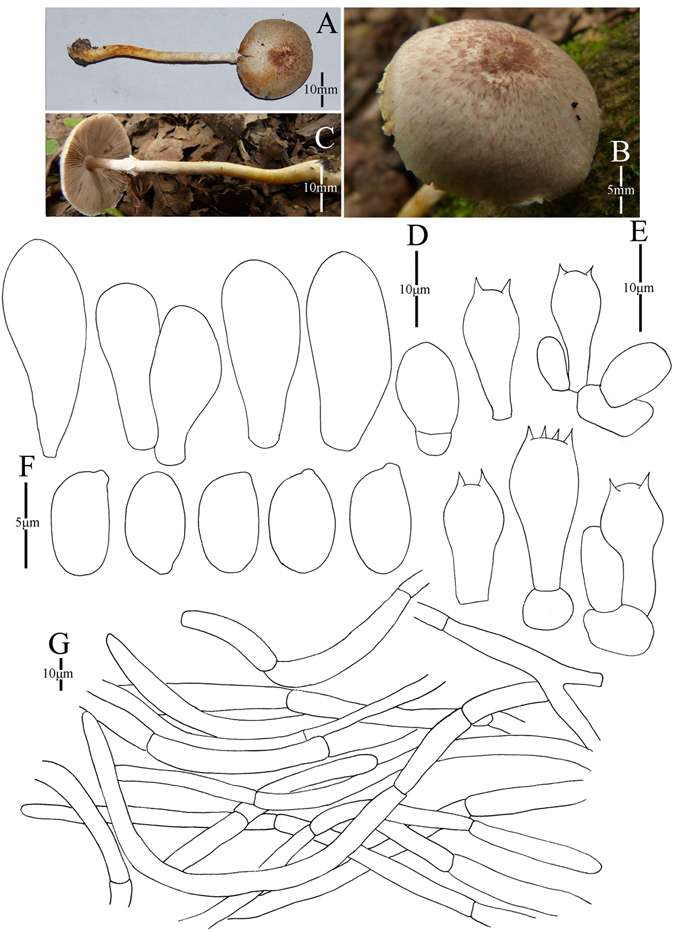
Morphology of *Agaricus parvibicolor* (**A**–**C**): Basidiome in field (*ZRL2012029*), (**D**): Cheilocystidia, (**E**): Basidia, (**F**): Basidiospores, and (**G**): Pileipellis hyphae.

2. *Agaricus parvibicolor* L.J. Chen, R.L. Zhao & K.D. Hyde, *Fungal Divers*. **72** (1): 1–197, 2015. Figure 21

*Pileus* 23 mm in diam.; convex, margin straight, slightly exceeding; surface dry, covered by fibrils on whole cap; fibrils broken into fibrillose scales at disc, purple, reddish brown, tiny, triangular, fading and scattered towards the margin. *Context* 2 mm thick, flesh, white. *Lamellae* 2–5 mm broad, free, crowded, broad, pink or pinkish brown first, then brown, edge even. *Annulus* up to 4 mm in diam., single, fragile, membranous, white, pendant, smooth on both sides. *Stipe* 60 × 2 (4 at base) mm, white, hollow, cylindrical, surface dry, fibrillose. Odour of almonds. Basidiome flavescent when touching, bruising and cutting.

KOH reaction: positive yellow. Schäffer’s reaction: positive, reddish orange on dry specimen.

*Basidiospores* 5.5–6.3 (7.1) × 3.4–3.9 μm, [x = 6.0 ± 0.4 × 3.6 ± 0.1, Q = 1.5–1.8, Q_m_ = 1.7 ± 0.1, n = 20], ellipsoid, elongate, smooth, thick-walled, brown. *Basidia* 13.3–17.9 × 6.1–7.5 μm, clavate, hyaline, 4-spored, smooth. *Cheilocystidia* 16.6–30 × 6–13.6 μm, smooth, mostly clavate, some broadly clavate, hyaline. *Pleurocystidia* absent. *Pileipellis* a cutis composed of hyphae of 3–6.7 μm in diam., smooth, cylindrical, light brown, slightly constricted at some septa.

Habitat: solitary on the side of road.

*Specimens examined*: Wuliang Mountain, Jingdong County, Yunnan Prov., China, collected by Tian Qing, *ZRL2012029* (HMAS275764).

Notes: *Agaricus parvibicolor* is originally described from Thailand, and characterized by its slender basidiome, reddish brown to violet brown fibrils on the pileus and simple cheilocystidia^19^. Our specimen has the same morphological characters with the original description except of the little larger basidiospores (5.2 × 3.3 μm^19^). In the phylogeny, our specimen also nests with the type specimen of *A*. *parvibicolor* under fully supports (Figs 1–2).

**Figure 22:**
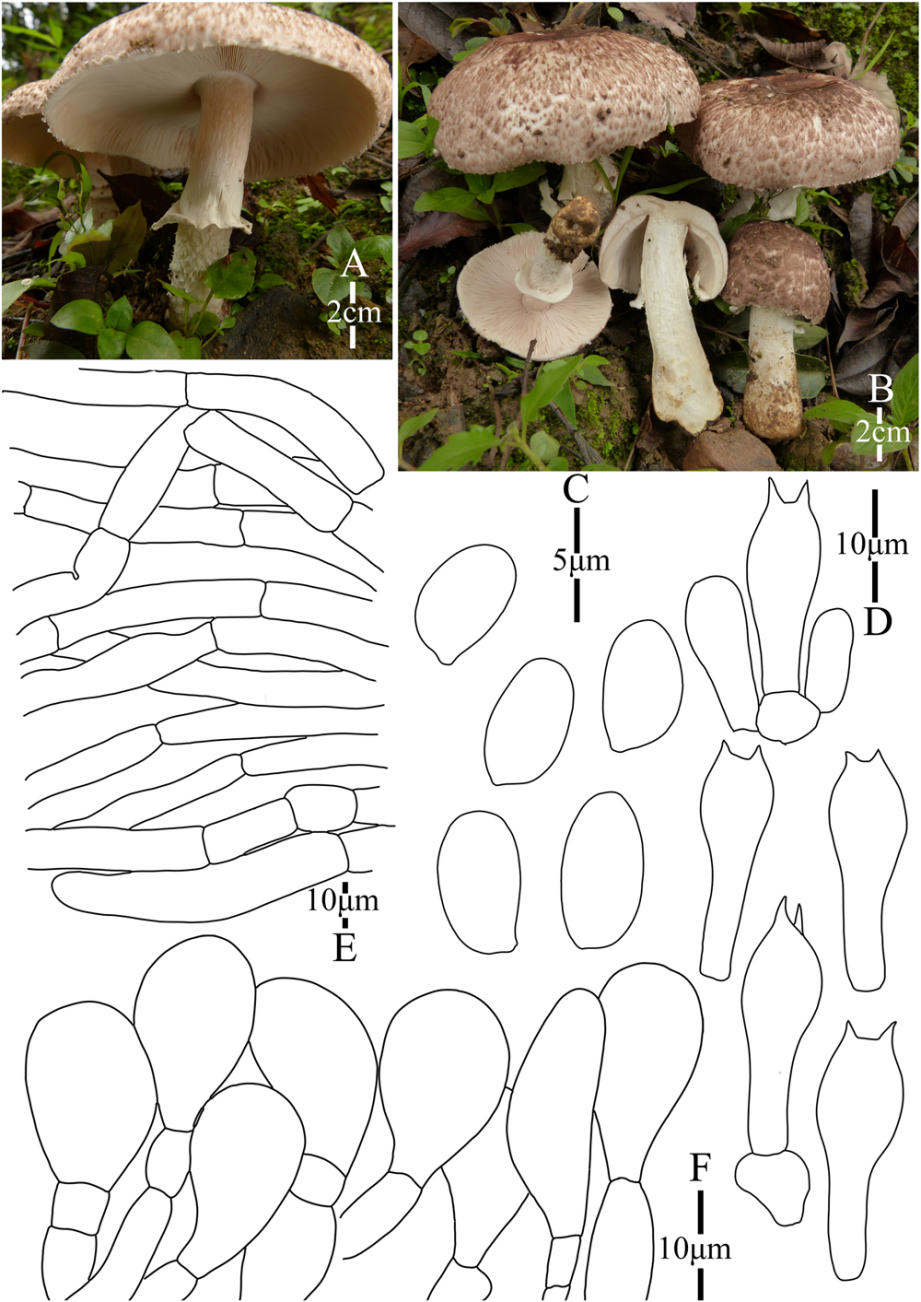
Morphology of *Agaricus megalosporus* (**A**,**B**): Basidiome in field (*ZRL2012199*), (**C**): Basidiospores, (**D**): Basidia, (**E**): Pileipellis hyphae, and (**F**): Cheilocystidia.

3. *Agaricus megalosporus* J. Chen, R.L. Zhao, Karunarathna & K. D. Hyde, *Cryptogam*., *Mycol*., **33** (2): 145–155, 2012. Figure 22

*Pileus* 50–100 mm in diam., parabolic with truncated top, then convex, slightly depressed at disc when mature; margin decurved, exceeding, with appendiculate remains of universal veil, crenate; surface dry, covered by fibrillose scales on the whole cap, appressed, brown or reddish brown, triangular. *Context* 7–8 mm thick, flesh, white. *Lamellae* 5–6 mm broad, free, crowded, narrow, white first, then pinkish, pinkish brown, brown in age, edge even. *Annulus* up to 16 mm in diam., single, membranous, white, pendant, upper side smooth, lower side heavily fibrillose. *Stipe* 60–100 × 7–12 (10–25 at base) mm, white, hollow, clavate with bulbous base, surface dry, floccose below the annulus. Odour of almonds. Basidiome flavescent when touching and bruising. Discoloration yellowish, then orange after several minutes on cutting.

KOH reaction: positive yellow. Schäffer’s reaction: positive, reddish orange on dry specimen.

*Basidiospores* 5.8–6.9 × 3.2–3.8 μm, [x = 6.3 ± 0.3 × 3.5 ± 0.2, Q = 1.6–1.9, Q_m_ = 1.8 ± 0.1, n = 20], ellipsoid, elongate, smooth, thick-walled, brown. *Basidia* 15.7–23 × 6.5–7.7 μm, clavate, hyaline, 4-spored, smooth. *Cheilocystidia* 15–24.5 × 7–16 μm, smooth, clavate to broadly clavate, 1–2 septa at base, hyaline. *Pleurocystidia* absent. *Pileipellis* a cutis composed of hyphae of 4.7–11.6 μm in diam., smooth, cylindrical, brown, slightly constricted at septa.

Habitat: scattered or gregarious on soil in forest.

*Specimens examined*: Lincang, Nanban Village, Cangyuan County, Yunnan Prov., China, collected by Philippe Callac, 10 July 2012, *ZRL2012199* (HMAS278044).

Notes: *Agaricus megalosporus* is firstly described from Thailand^18^. It has middle to large sized basidiome and larger basidiospores compared with other species of section *Minores*. Our specimen has almost all identical morphological characters with the original description, except of the terminal element of the cheilocystidia from this study is slightly wider than those of original description^18^. Furthermore our specimen has the identical ITS sequence with those of type specimen. Considering no more significant difference between them, we identified our specimen as *A*. *megalosporus*, which is new records for China.

## Materials and Methods

### Sampling

Species of *Agaricus* subgenus *Minores* and its sister subgenus *Minoriopsis* reported in previous studies are included in this study^1, 4, 10, 18, 42–45^. In total, 305 sequences from temperate Europe, tropic Asia, Africa, Oceania and America were retrieved from GenBank. A further 100 new collections of *Agaricus* section *Minores* from China were made and included (Supplementary Table 1), of which 19 are tropical, 46 are subtropical, and 16 are temperate^48^; and 19 are separated as Tibetan Plateau in this study because of their extreme living environment.

### Morphological examination

Every newly collected specimen was photographed *in situ*. Macro-morphological characteristics and biochemical color reactions were recorded from fresh specimens. The specimens were dried in a food drier at 60 °C. Anatomical and cytological features including basidiospores, basidia, cystidia and pileipellis were observed under a Olympus CX31 microscope. At least 20 measurements were made. Data were recorded as follows: X = the mean of length by width ± SD; Q = the quotient of basidiospore length to width, and Q_m_ = the mean of Q values ± SD. The protocol of morphological study and chemical reaction followed Largent’s methodology^49^. Specimens are deposited at Herbarium Mycologicum Academiae Sinicae (HMAS).

### DNA extraction, PCR amplification and sequencing

Total genomic DNA was extracted from dry specimens using an E.Z.N.A. Forensic DNA Extraction Kit (D3591-01, Omega Bio-Tek) and followed the manufacturer’s protocol. The internal transcribed spacer (ITS) was amplified with the PCR primers ITS4/ITS5, nuclear large ribosomal subunit (LSU) with primers LR5/LROR, translation elongation factor (tef1-α) with primers 983 f/1567r and RNA polymerase II gene (rpb2) with 6 F/7CR^50–53^. PCR amplification was performed with 3 μl primer (1.5 μl each), 25 μl 2xPower Taq PCR MasterMix, 20 μl ddH_2_O and 2 μl DNA template. The PCR procedure for ITS and LSU regions were as follows: Initial denaturation for 3 minutes at 94 °C; 35 cycles of denaturation for 1 minute at 94 °C; annealing for 50 seconds at 52 °C; extension for 1 minute at 72 °C; and finally left at 72 °C for 10 minutes. The tef1-α region amplification involved initial denaturation at 94 °C for 3 minutes, 40 cycles of denaturation at 94 °C in 50 seconds, annealing at 55 °C in 60 seconds, extension at 72 °C for 80 seconds and finally left at 72 °C for 10 minutes. The rpb2 region amplification involved an initial denaturation for 1 minute at 95 °C, denaturation at 95 °C for 1 minute, annealing at 55 °C for 1 minute, extension at 72 °C for 1 minute and increase to 1 second per cycle, for 34 cycles and finally left at 72 °C for 10 minutes^50–53^. The PCR products were sent to a commercial company for sequencing. Sequencing was performed in two directions for each gene to ensure accuracy.

### Phylogenetic analysis

A total of 534 sequences are presented in this study. The multi-gene dataset contained 154 assembled sequences, comprising 154 ITS, 128 LSU, 123 tef1-α and 73 rpb2 sequences. Among them, 334 are new contributions from this study, while 200 were download from GenBank (Supplementary Table 1). Sequences are assembled in Geneious 9.0.2., aligned in BioEdit V.7.0.4^54^ and adjusted manually to exclude the ambiguous regions. The alignment has been submitted to TreeBase (submission ID: 20505). Bayesian Inference (BI) analysis was performed in MrBayes 3.1.2^55^. The best substitution model for ITS, LSU, tef1-α and rpb2 regions were inferred by MrModeltest2.2^56^: GTR + I + G for ITS, LSU and rpb2; SYM + I + G. for tef1-α. Ten million generations were run for six Markov chains, and sampled every 100th generation resulting in 100,000 trees. Burn-ins was determined in Tracer v1.6 with effective sample sizes (ESS) of 200 or higher (http://tree.bio.ed.ac.uk/software/tracer). Those trees sampled prior to searches reaching a split deviation frequency value reaching 0.01 were discarded as the burn-in, and the remaining trees were used to calculate Bayesian posterior probabilities (PP). Maximum likelihood (ML) analysis was performed in raxmlGUI 1.5b1 with GTRGAMMA model with 1000 replicates^57^. Maximum parsimony analysis was performed in PAUP*4.0b 10^58^. One thousand heuristic searches were conducted with random sequence addition, tree bisection-reconnection (TBR) branch swapping and gaps were treated as missing data. Parsimony bootstrap values were obtained from 1000 bootstrap replicates, with starting trees obtained via stepwise addition, random sequence addition, and max-trees set to 1,000.

### Calibration strategies and age estimation of clades within Agaricus section Minores

The age of 30 Ma of *Agaricus* subgenus *Minores* is cited from our previous study^9^, which was conducted from a fossil-calibrated analysis referred from two Agaricomycetes fossils: *Archaeomarasmius leggetti* Hibbett, D. Grimaldi & Donoghue, as representative of the minimum age of 90 million years ago of *Agaricales* ^59^, and *Quatsinoporites cranhamii* S.Y. Sm., Currah & Stockey as representative of the minimum age of 113 million years ago of *Hymenochaetales* ^60^. The nrITS, nrLSU, tef1-α and rpb2 datasets of *Agaricus* subgenus *Minores* + *Agaricus subgenus Minoriopsis* + outgroup *Agaricus campestris* are aligned using MUSCLE v3.6^61^ separately.

Divergence times were estimated using BEAST v1.8^62^. A XML file was constructed with BEAUTI v1.8. Per-gene alignments were imported as separate partitions. Clock and substitution models were set to be unlinked (independently estimated for each gene partition). As substitution models, we used the GTR for rpb2, and HKY for nrITS, nrLSU and tef1- respectively, based on jModelTest v2^62^. We used the uncorrelated lognormal relaxed clock model^63, 64^, specifying a gamma distribution for the ulcd.mean parameter with a shape of 1.0, scale of 0.001, and offset 0. On the calibrated nodes, we specified a prior gamma distribution with an arbitrarily long tail (scale of 4) and offset ages of 30 for *Agaricus* subgenus *Minores* ^9^. We ran Monte Carlo Markov Chains of 50 million generations, logging states every 5000 generations. We compared the log files of each run in Tracer v1.6^65^, evaluating convergence and mixing, ensuring that Effective Sample Sizes were at least 200. An ultrametric maximum-clade-credibility (MCC) tree was summarized using TreeAnnotator 1.8, discarding 10% of states as burn-in and annotating clades with ≥0.8 posterior probability.

## Electronic supplementary material

Supplementary Information
